# Cultural Dynamics in the Levantine Upper Paleolithic, ca. 40–33 ky BP: Insights Based on Recent Advances in the Study of the Levantine Aurignacian, the Arkov-Divshon, and the Atlitian

**DOI:** 10.1007/s41982-024-00176-0

**Published:** 2024-04-29

**Authors:** Maayan Shemer, Omry Barzilai, Ofer Marder

**Affiliations:** 1 Department of Archaeology, Ben-Gurion University of the Negev, P.O. Box 653, 84105 Beer Sheva, Israel; 2 Archaeological Research Department, Israel Antiquities Authority, The National Campus for the Archaeology of Israel, 1 Nahman Avigad Street, 9370726 Jerusalem, Israel; 3 The Leon Recanati Institute for Maritime Studies, University of Haifa, Mount Carmel, 3498838 Haifa, Israel

**Keywords:** Atlitian, Arkov-Divshon, Levantine Aurignacian, Upper Paleolithic, Cultural dynamics, Migration patterns

## Abstract

The chrono-cultural sequence of the Levantine Upper Paleolithic went through several major revisions during approximately a century of focused research, each revision contributing to shedding light on the mosaic of cultural entities and the complex social and cultural dynamics composing the Levantine Upper Paleolithic. The current state of research suggests the co-inhabitance of two cultural groups: the Early Ahmarian and the Levantine Aurignacian. Two other cultural entities, the Arkov-Divshon and the Atlitian, are regarded as younger manifestations and were tentatively suggested to relate to the Levantine Aurignacian. This paper presents a research synthesis of two case studies: Manot Cave, located in western Galilee, Israel, and Nahal Rahaf 2 Rockshelter in the Judean Desert. The application of high-resolution excavation methods, alongside detailed documentation of the stratigraphy and site-formation processes and wide-scale radiocarbon-based absolute dating, marked these sites as ideal for chrono-cultural study through the analyses of flint industries. The results indicate a clear distinction between the Levantine Aurignacian and the Arkov-Divshon/Atlitian industries and a chronological overlap between the Arkov-Divshon, Levantine Aurignacian, and possibly with the Early Ahmarian. Subsequently, we suggest another revision of the currently accepted chrono-cultural model: not two, but at least three cultural entities co-inhabited the Levant at ca. 40–30 ky cal BP. This study further suggests an evolvement of the Atlitian flint industries from the Arkov-Divshon and stresses the foreign cultural features of the Levantine Aurignacian. These results were used to construct an updated model of migration and possible interaction patterns.

## Introduction

The Levant has long been a focus of prehistoric research due to two key elements: its geographic position, constituting a land conduit between Africa and Eurasia, and the known presence of both Neanderthal and modern human populations during the Middle Paleolithic period (e.g., Akazawa et al., 1995; Arensburg & Tiller, 2019; Bar-Yosef et al., 2019; Been et al., 2017; Bergman & Stringer, 1989; Hershkovitz et al., 2015, 2018; Hovers et al., 1995; Jelinek, 1982; Mellars, 2004; Solecki, 1975; Stringer et al., 1989; Valladas et al., 1987; Vandermeersch, 1981, 2007; Vandermeersch & Bar-Yosef, 2019). Preliminary documentations of Levantine prehistoric cultural composition were conducted in the early twentieth century by pioneer European researchers as a part of wide, regional studies (e.g., Ewing, 1947; Garrod, 1934; Garrod & Bate, 1937; Neuville, 1934, 1951; Turville-Petre, 1927, 1932; Zumoffen, 1908). These formed the basis for the first chrono-cultural models of the region, relying primarily on the comparative characterization of lithic industries (e.g., Garrod, 1953, 1957; Neuville, 1934, 1951).

The initial chrono-cultural model of the Levantine Upper Paleolithic period (ca. 50–23 ky cal BP), established by Neuville (1951) and Garrod (1953, 1957), encompassed six phases: Phase I, termed the “Emiran,” was conceived as a transitional phase, reflecting a step in the development of Middle Paleolithic traditions into Upper Paleolithic industries (e.g., Garrod, 1951, 1955, 1957; Turville-Petre, 1932). Phase II remained unnamed due to its limited presence and the small size of the affiliated collections (Garrod, 1957; Gilead, 1991; Neuville, 1951). Phase III was characterized by the presence of elements commonly associated with the European Aurignacian culture, namely, carinated scrapers and osseous artifacts. These appeared together with an abundance of pointed blades, initially referred to as Font-Yves points and later granted a local, more commonly used nomenclature of “el-Wad points” (e.g., Garrod, 1953, 1957; Garrod & Bate, 1937; Gilead, 1991). Phase IV showed an increase in the dominance of Aurignacian components, whereas el-Wad points became rare (Garrod, 1953, 1957). Phase V, named the “Atlitian,” was characterized by the abundance of burins and “narrow” (i.e., lateral) carination and was suggested as representing a specialized evolvement of Phase IV (Garrod, 1953, 1957). The youngest Phase VI, named “Kebaran,” encompassed full-fledged microlithic industries dominated by pointed and truncated bladelets (Bar-Yosef, 1970; Garrod, 1953, 1957; Neuville, 1951).

The six-phase model formed the basis for reconstructing a continuous, linear cultural evolution, encompassing all of the lithic industries from the Middle Paleolithic to the Epipaleolithic of the southern Levant. The presence of Aurignacian elements in Phases III–IV was perceived as a European influence on the local industries (Garrod, 1957). The paucity of osseous artifacts, and particularly of antler-made, split-based points that were hallmarks of European Aurignacian assemblages in the early twentieth century, alongside the presence of el-Wad points, led to the distinction of the Levantine Phases III–IV from the Aurignacian of Europe, ascribing to them a local nomenclature: Lower and Upper Antelian (for Phases III and IV, respectively; Copeland & Hours, 1971; Garrod, 1957). Only later was the possibility that these assemblages reflect actual European populations established, providing a first indication of a “back migration” from Eurasia to the Levant and coining the term “Levantine Aurignacian” (e.g., Belfer-Cohen & Bar-Yosef, 1981; Besançon et al., 1975; Copeland, 1975; Hours, 1974; and see a similar suggestion that was later withdrawn in Garrod, 1953).

Further modifications to the Levantine Upper Paleolithic chrono-cultural model were introduced in the second half of the twentieth century. Accumulation of data from new survey and excavation projects (e.g., Bar-Yosef & Phillips, 1977; Gilead, 1993; Gilead & Bar-Yosef, 1993; Marks, 1976a, 1977a, 1983), alongside comprehensive studies of the lithic industries from key sites in the Mediterranean and arid regions of the southern Levant (e.g., Belfer-Cohen, 1980; Gilead, 1981; Goring-Morris, 1987), showed marked differences between Phases III and IV. These led to a reconsideration of the cultural affiliation of Phase III and to the realization that this phase consists of two distinct components: one containing Levantine Aurignacian hallmarks and another encompassing a very blade-oriented flint industry that was named “Ahmarian” (e.g., Gilead, 1981, 1991; Marks, 1981, 2003). In contrast to the Levantine Aurignacian, the Ahmarian was, and at large still is, considered to represent indigenous populations, encompassing local lithic traditions that evolved from earlier Paleolithic industries (e.g., Belfer-Cohen & Goring-Morris, 2018; Gilead, 1981, 1991; Kuhn, 2004; Marks, 1981, 2003; Williams, 2003). Most significantly, however, the archaeological data accumulated by the end of the twentieth century indicated the co-existence of the Ahmarian and the Levantine Aurignacian in the Levant ca. 40–30 ky BP, leading to a revision of the linear cultural evolution approach. Instead, a new model was established: The “Two Traditions” or “Parallel Phyla” model (e.g., Bar-Yosef & Belfer-Cohen, 1996; Belfer-Cohen & Bar-Yosef, 1981; Gilead, 1981, 1991; Goring-Morris, 1980; Marks, 1981, 2003). The assumption that some degree of contact between two populations sharing a geographic region was inevitable led to further investigation into the manifestations of such contacts within the archaeological record, trying to extrapolate on aspects of social behaviors (e.g., Gilead, 1991; Goring-Morris & Belfer-Cohen, 2003, 2018; Lengyel, 2007; Marks, 2003; Williams, 2003; Williams & Bergman, 2010).

At the end of the twentieth century, a division of the Levantine Upper Paleolithic into three primary stages was established: Initial, Early, and Late (e.g., Bar-Yosef & Belfer-Cohen, 2010; Belfer-Cohen & Goring-Morris, 2003, 2014, 2017). The Initial Upper Paleolithic (IUP; ca. 50–45 ky cal BP; Barzilai, 2022; Boaretto et al., 2021) correlates to Phase I of the six-phase model. It includes an early manifestation, the Emiran, and a late manifestation corresponding to “Boker Tachtit, Level 4” (e.g., Barzilai, 2022; Boaretto et al., 2021; Fox & Coinman, 2004; Goder-Goldberger et al., 2023; Kadowaki et al., 2019; Kuhn, 2019; Marks & Ferring, 1988). While still considered to represent a techno-typological “middle phase” between the earlier Middle Paleolithic and the later Early Ahmarian industries, the origin of these entities has been a subject of recent discussion, as some scholars suggest that the initial emergence of IUP industries was outside the Levant; thus, their presence in the region marks the arrival of new populations (e.g., Barzilai, 2022; Rose & Marks, 2014; Wurz & Van Peer, 2012; but see, e.g., Goder-Goldberger & Malinsky-Buller, 2022; Goder-Goldberger et al., 2020; Goring-Morris & Belfer-Cohen, 2020; Meignen, 2012, for an opposite view, arguing for continuance in certain attributes of the lithic industries, and therefore for a local evolvement).

The second stage, the Early Upper Paleolithic (henceforth EUP; ca. 45–34/32 ky cal BP, e.g., Gilead, 1991) encompasses both Phases III and IV of the six-phase model, namely, the Early Ahmarian and the Levantine Aurignacian. The Early Ahmarian is best characterized by associated flint industries, which are predominately oriented towards a systematic production of pointed blades from narrow-fronted cores (e.g., Goring-Morris & Davidzon, 2006). Tool typology shows that these blades were used for various purposes, primarily as possible hunting points with the application of minor shaping near the edge (el-Wad points) or for other activities, as endscrapers and other informal tools (e.g., Bar-Yosef & Phillips, 1977; Bergman, 1988; Davidzon & Goring-Morris, 2003; Gilead, 1981; Gilead & Bar-Yosef, 1993; Goring-Morris & Davidzon, 2006; Jones et al., 1983; Kuhn et al., 2009). The Early Ahmarian flint industry includes two primary facies: A Northern facies, found primarily in the Mediterranean region, which focused on a bi-directional blade reduction technique, and a Southern facies, found in the arid regions of the Levant, characterized by its reliance on uni-directional blade reduction (e.g., Abulafia et al., 2021; Bergman, 1988; Goring-Morris & Belfer-Cohen, 2018; Goring-Morris & Davidzon, 2006; Kadowaki et al., 2015; Kuhn et al., 2009; Marks, 2003; Tostevin, 2012). Absolute chronology indicates a general age range of ca. 47–35.5 ky cal BP for the Early Ahmarian, albeit results tend to fall into two main clusters: ca. 47–43 ky cal BP (Alex et al., 2017; Bosch et al., 2015; Rebollo et al., 2011) and ~ 39.0–35.5 ky cal BP (Douka et al., 2013; Gilead & Bar-Yosef, 1993; Kuhn et al., 2009). At its peak, the Early Ahmarian presented a wide geographic distribution, encompassing wide and various ecotones. It is considered to represent the technologies and ideas of populations indigenous to the Levant (e.g., Bar-Yosef & Belfer-Cohen, 2010; Belfer-Cohen & Goring-Morris, 2003, 2017; Gilead, 1991; Kuhn et al., 2009; Marks, 1981, 2003).

The Levantine Aurignacian is best characterized by its rich osseous industries and diversified flint reduction approaches. Animal remains were utilized for the production of domestic tools as well as mobile art and decorative items, including the targeted use of antlers for weaponry (e.g., Bar-Yosef & Belfer-Cohen, 2010; Belfer-Cohen & Bar-Yosef, 1981; Newcomer & Watson, 1984; Tejero et al., 2016, 2018, 2021). The flint assemblages reflect the use of multiple, distinct reduction sequences for the production of large blades, flakes, and bladelets, with the former possibly produced off-site (e.g., Bar-Yosef & Belfer-Cohen, 2019; Belfer-Cohen & Bar-Yosef, 1981; Lengyel, 2007; Shemer et al., 2024; Shimelmitz et al., 2018; Williams, 2003; Williams & Bergman, 2010). Absolute chronology, based primarily on the sequences from Ksâr ‘Akil Rockshelter, Kebara, and Manot Caves, indicates a general age range of ca. 38–34/33 ky cal BP (Alex et al., 2017; Bar-Yosef et al., 1996; Bosch et al., 2015; Douka et al., 2013; Shemer et al., 2024), with more limited ranges of ca. 37.5–36.0 ky cal BP recently suggested based on radiocarbon ages from Manot Cave, Area E (Alex el al., 2017; Shemer et al., 2024). The geographical distribution of the Levantine Aurignacian was confined primarily to the Mediterranean parts of the region (e.g., Bar-Yosef & Belfer-Cohen, 2010, 2019; Belfer-Cohen & Goring-Morris, 2003; Gilead, 1991; Marks, 1981). Based on multiple shared attributes in flint reduction techniques and tool typology, as well as in osseous weaponry and decorative items, the Levantine Aurignacian is widely considered an indication of a “back migration,” representing the dispersal of Eurasian populations and ideas into the region (e.g., Bar-Yosef & Belfer-Cohen, 1996, 2010; Belfer-Cohen & Goring-Morris, 2003, 2017; Copeland, 1975; Gilead, 1991; Hours, 1974; Marks, 1981).

The Late Upper Paleolithic (henceforth LUP; ca. 34/32–23 ky cal BP, e.g., Gilead, 1991) is less well-known, presenting a high degree of ambiguity regarding the sets of attributes, general processes, and cultural entities that define it. Three cultural entities were traditionally associated with the LUP: The Atlitian (Phase V of the six-phase model), found primarily in the Mediterranean region; the Arkov-Divshon, a desert-adapted entity that was broadly defined at the end of the twentieth century based on sites in the Negev, Sinai, and southern Jordan; and the Masraqan, often referred to as “Late Ahmarian” (e.g., Bar-Yosef & Belfer-Cohen, 2010; Belfer-Cohen & Goring-Morris, 2003; Gilead, 1991; Kadowaki et al., 2015; Marks, 1981, 2003). However, all three suffered from broad techno-typological descriptions and poorly established chronology that impeded a comprehensive, comparative characterization (e.g., Bar-Yosef, 2006; Belfer-Cohen & Goring-Morris, 2003, 2018; Goring-Morris & Belfer-Cohen, 2020; Shemer et al., 2023; Williams, 2003, 2006).

Marked differences between lithic industries associated with the Atlitian and Arkov-Divshon on the one hand and the Ahmarian on the other led to the distinction of the former two from the latter, primarily based on the absence of systematic blade reduction and the lack of blade predominance. Instead, the possible association of the Arkov-Divshon and the Atlitian with the Levantine Aurignacian was tentatively suggested (e.g., Gilead, 1981, 1991; Marks, 1981, 2003), somewhat deferring to Garrod’s initial suggestion of specialized evolvement (Garrod, 1953, 1957). However, a secure connection was never fully established due to wide variability in some of the key attributes associated with each cultural entity. Table 1 presents a summary of characteristic features of the flint assemblages from primary occupation layers associated with the three cultural entities, demonstrating this issue. Some of the variability was addressed, ascribed to differences in excavation and processing methodology (i.e., in el-Wad and el-Khiam), to the incompleteness of the examined assemblages (i.e., in Yabrud and Ksâr ‘Akil), to the effect of post-depositional erosion (i.e., in Arkov and possibly Ramat Materd I), and to possible local adaptation and differences in site function (i.e., in Nahal Ein Gev I and Fazael IX, e.g., Belfer-Cohen et al., 2004; Ghazi, 2013; Shemer et al., 2023; Williams, 2003). As an alternative, the Arkov-Divshon and the Atlitian were grouped under the general description of “flake-based industries” of the Levantine Upper Paleolithic (e.g., Bar-Yosef & Belfer-Cohen, 1996; Belfer-Cohen & Goring-Morris, 2003, 2018; Gilead, 1981, 1991; Marks, 1981, 2003; Williams, 2003).

**Table 1:** Summary of characteristic features of the flint assemblages associated with the Levantine Aurignacian, Arkov-Divshon, and Atlitian from primary sites in the southern Levant. *Calculated based on published data. *n/a* data not available. Italics font marks the case studies at the base of this research. Average values are presented for multi-layered occurrences

	Cultural affiliation	% of bladelets from the debitage/tools	% of blades from the debitage/tools	% of frontal carination from the tools	% of lateral carination from the tools	% of burins on truncation from the tools	Burins/Endscrapers ratio	Aurignacian Index	Data source
**Ksâr ‘Akil** **Phase 5**	Levantine Aurignacian	17.0/n/a	27.5/n/a	n/a	n/a	3.6*	1:7	45.1	Williams & Bergman, 2010
***Manot Cave*** ***Phase 2***	*Levantine Aurignacian*	*19.5/21.5*	*3.4/26.4*	*11.9*	*2.9*	*0.8*	*1:1.5*	*21.1*	Shemer et al., 2024
**Hayonim Cave Layer D**	Levantine Aurignacian	12.7/n/a	9.7/n/a	12.9	0.6	6.2	1:2.3	21.2	Belfer-Cohen & Bar-Yosef, 1981
**Kebara Cave** **Layers II–I**	Levantine Aurignacian	9.3/19.0	17.5/20.5	14.7*	1.0*	3.6	1:2.1	24.6	Bar-Yosef & Belfer-Cohen, 2019
**Raqefet Cave** **Layers III, Area B–G/18–23**	Levantine Aurignacian	4.4/5.4	6.5/23.1	12.0	1.1	1.8	1:2.7	n/a	Lengyel, 2007
**Sefunim Cave** **Layers 8–10 (**Ronen, 1984**, correlating to Layer V in **Shimelmitz et al., 2018**)**	Levantine Aurignacian	9.4/7.0	35.2/43.1	15.9	0.8	5.2	1:2.5	18.1*	Ronen, 1984: 231–274
***Nahal Rahaf 2***	*Arkov-Divshon*	*23.1/42.7*	*1.5/8.1*	*1.4*	*14.7*	*0.4*	*1:1.6*	*10.8*	Shemer et al., 2023
**Ein Aqev (D31)**	Arkov-Divshon	23.4/13.1	20.1/29.1	2.3	2.4	4.6	1:0.6	n/a	Marks, 1976b
**Arkov (D22)**	Arkov-Divshon	10.2/5.2	18.8/24.7	6.2	2.4	1.3	1:0.8	n/a	Williams, 2003
**Ramat Matred I**	Arkov-Divshon	n/a	n/a	0.2	11.2	4.9	1:0.3	12.6*	Gilead, 1993
**Har Horsha I**	Arkov-Divshon	n/a	n/a	4.8	5.0	1.7	1:3	14.0*	Belfer-Cohen & Goring-Morris, 1986
**Palm View I (Areas 3–4)**	Arkov-Divshon	23.9/n/a	20.3/n/a	6.6*	19.8*	4.7*	1:3.7	n/a	Schyle, 2015
**Ksâr ‘Akil** **Phase 6**	Atlitian	48/n/a	21.0/n/a	14.2	16.8	11.5	1:0.5	12.2	Williams & Bergman, 2010
**Yabrud II** **Layer 1**	Atlitian	n/a/17.8*	n/a/34.4*	6.2*	4.0*	1.1*	1:1.9*	n/a	Ghazi, 2013
***Manot Cave*** ***Phase 1***	*Atlitian*	*20.6/43.3*	*2.8/5.5*	*2.3*	*7.1*	*4.2*	*1:0.6*	*9.1*	Shemer et al., 2024
**El-Wad Cave** **Layer C**	Atlitian	n/a	n/a	n/a	n/a	n/a	1:0.8	n/a	Garrod & Bate, 1937
**El- Khiam Terrace Layer E (Nivel 9)**	Atlitian	n/a	n/a	4.0	n/a	5.2	n/a	4.9	González Echegaray, 1964
**Nahal Ein Gev I**	Atlitian	10.1/9.6	6.7/18.5	1.8*	n/a	26.2*	1:0.2	n/a	Belfer-Cohen et al., 2004
**Fazael IX**	Atlitian	n/a	n/a	n/a	n/a	54.1*	1:0.1	n/a	Goring-Morris, 1980

The Late Ahmarian/Masraqan, on the other hand, was related to the Early Ahmarian based on the typical use of uni- or bi-directional technique to produce bladelets from narrow-fronted cores. These reduction approaches were suggested to bear a close resemblance to the ones applied for blade reduction in the Early Ahmarian, albeit their aim for a smaller product (i.e., bladelets, rather than blades; Belfer-Cohen & Goring-Morris, 2003; Ferring, 1988; Goring-Morris, 1980, 1995; Kadowaki, 2013; Kadowaki et al., 2015; Marks, 2003; Williams & Bergman, 2010). The few absolute ages available suggest a range of ca. 33/30–20 ky cal BP, placing the Late Ahmarian/Masraqan at the very end of the Levantine Upper Paleolithic and at the threshold of the Epipaleolithic (Goring-Morris & Belfer-Cohen, 2003: appendix; Kadowaki, 2013; Nadel et al., 1995). Its resemblance to the Early Ahmarian is commonly regarded as an indication of technological continuity and local evolvement into microlithic industries (e.g., Goring-Morris & Belfer-Cohen, 2017; Kadowaki, 2013; Marks, 2003; Monigal, 2002; Nadel, 2003; Williams, 2003).

**Fig. 1:**
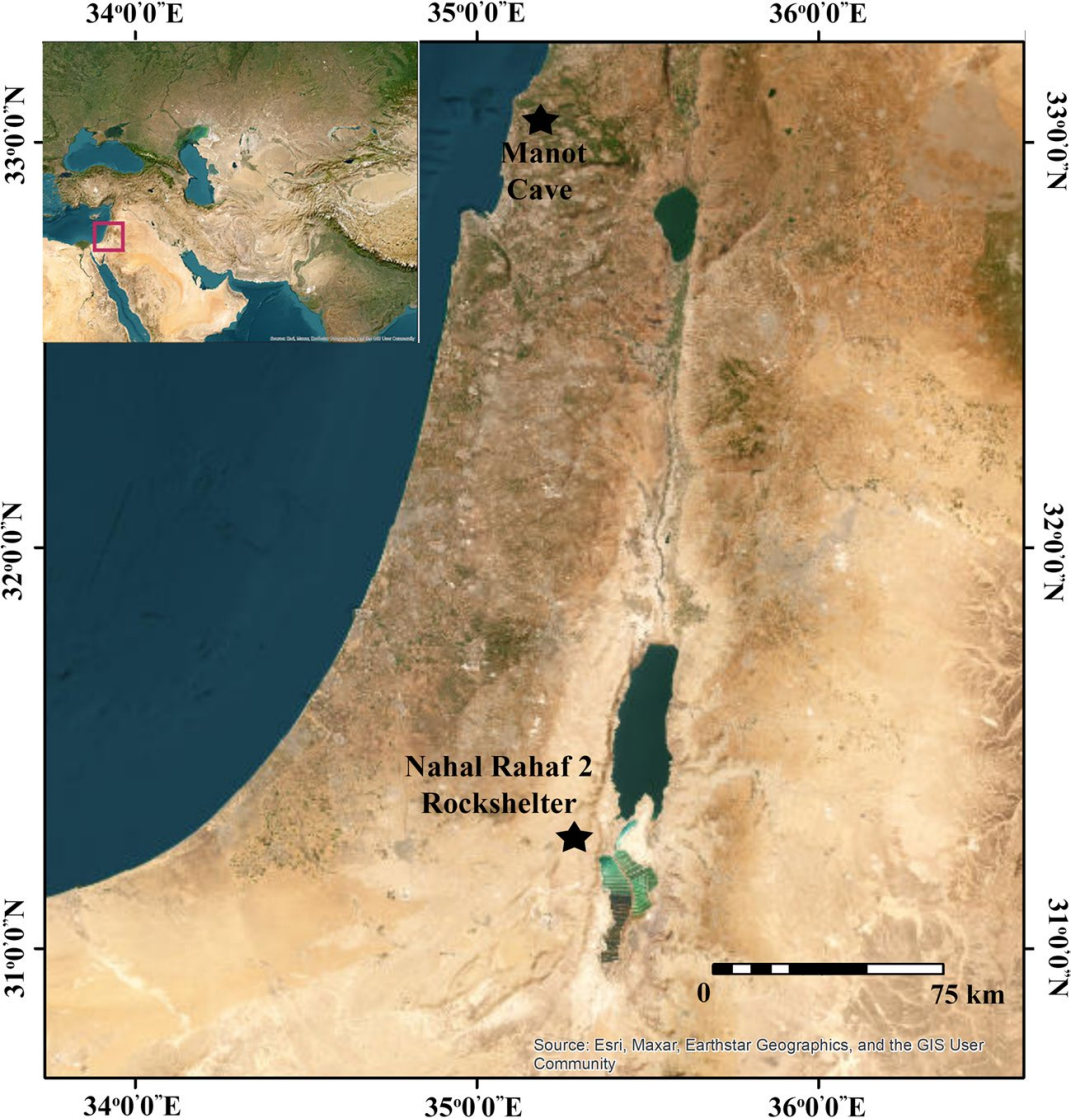
Geographic location of Manot Cave and Nahal Rahaf 2 Rockshelter

This paper encompasses the research synthesis of a study conducted in the recent years that examined the archaeological evidence for a possible connection between the Levantine Aurignacian and the “flake-based” industries of the Levantine Upper Paleolithic. It was based on two case studies: Manot Cave in the Levantine Mediterranean region and Nahal Rahaf 2 Rockshelter in the Judea Desert (Fig. 1). The two sites contain well-preserved stratified sequences ascribed to the Levantine Aurignacian and the Atlitian at Manot Cave, and Arkov-Divshon at Nahal Rahaf 2 Rockshelter (Shemer et al., 2023, 2024). The study focused on the characterization of lithic industries, combining radiocarbon chronology and considering high-resolution stratigraphy and site formation processes. These were used as the basis for chrono-cultural affiliations. The complete analyses of each of the case studies were published separately (Shemer et al., 2023, 2024) and are summarized here. These provided the database for the suggestions and ideas presented in this paper. The data collected from each of the case studies were tested here from a regional perspective. As a result, the current study suggests another revision of the Levantine model of cultural dynamics in the Upper Paleolithic period. Notably, however, issues regarding the Levantine Ahmarian industries are beyond the scope of this study and, therefore, presented here only in broad lines.

## The Case Studies and Their Contribution to the Study of Levantine Upper Paleolithic

### Nahal Rahaf 2 Rockshelter and the Arkov-Divshon Industries of the Arid Regions

The first case study, Nahal Rahaf 2 Rockshelter (henceforth NR2), is located in the Judean Desert, ca. 5 km west of the Dead Sea and ca. 90 km east of the Mediterranean shoreline. The rockshelter is composed of a large, partly roofed open space (ca. 35 m^2^) and two additional inner chambers with unknown extents that are currently completely filled with sediments. Excavations on site revealed ca. 1.5 m of deposits, including four occupation layers and three in situ combustion features that were associated with the Arkov-Divshon layers, together forming ca. 80 cm of archaeological accumulations (Barzilai et al., 2020; Marom et al., 2022; Shemer et al., 2023). The hyper-arid conditions in the Judean Desert were favorable for the preservation of organic remains, and accordingly, in addition to rich flint assemblages, the site yielded numerous animal bones and charcoal fragments. These attributes made NR2 a high-quality case study, providing a unique insight into a cultural entity whose definition and chronology were not well established, having relied primarily on surface collections with poor organic preservation (e.g., Baruch & Bar-Yosef, 1986; Belfer-Cohen & Goring-Morris, 1986; Ferring, 1976; Gilead, 1993; Marks & Ferring, 1976; Marks, 1976b; Schyle & Richter, 2015; Schyle & Uerpmann, 1988; Williams, 2003).

**Fig. 2:**
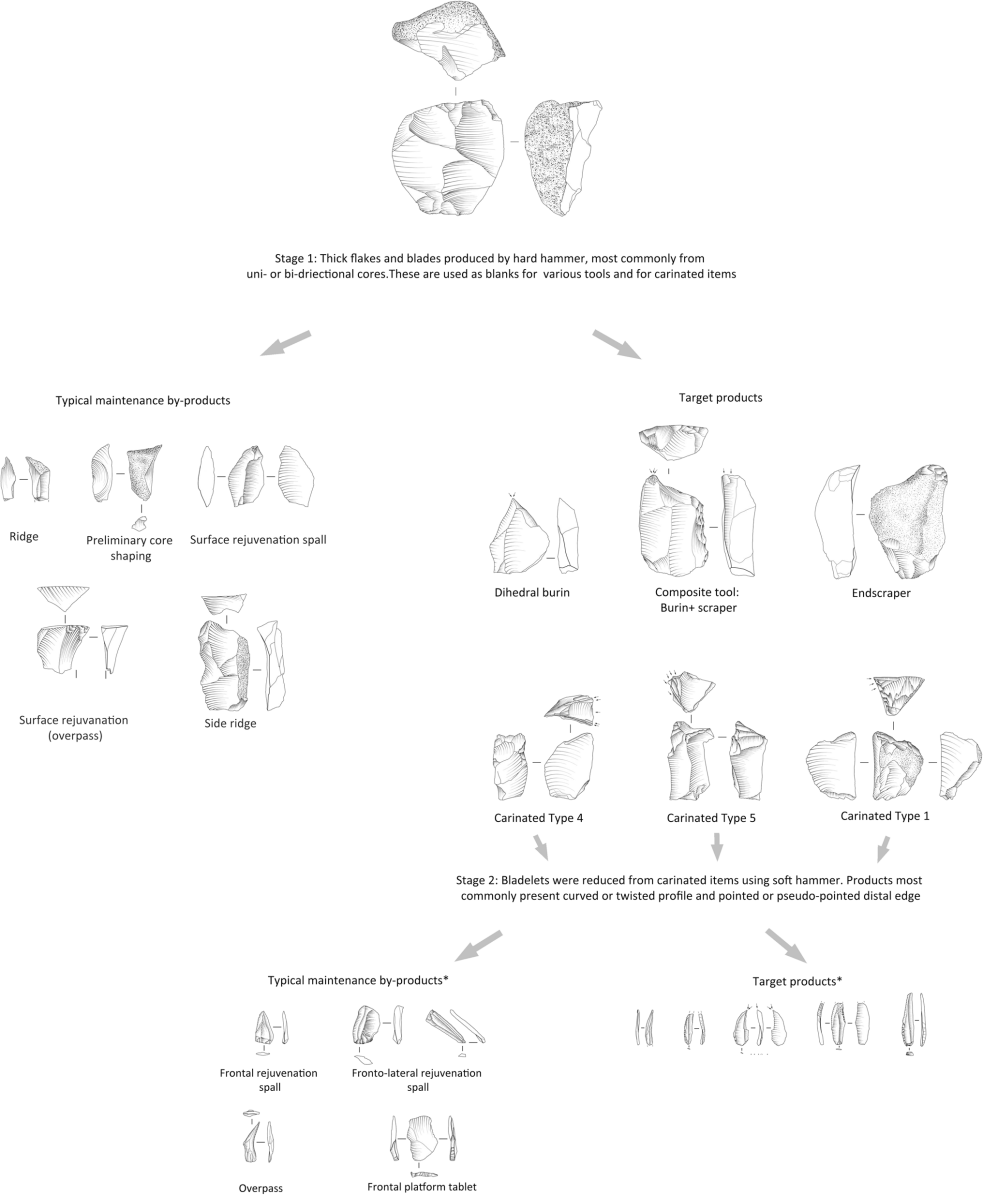
Schematic illustration of the two-stage reduction sequence characterizing the Arkov-Divshon industry, reconstructed based on refitted sequences and techno-typological attributes of the flint assemblages from Nahal Rahaf 2 Rockshelter (after Shemer et al., 2023: Fig. 10)

**Fig. 3:**
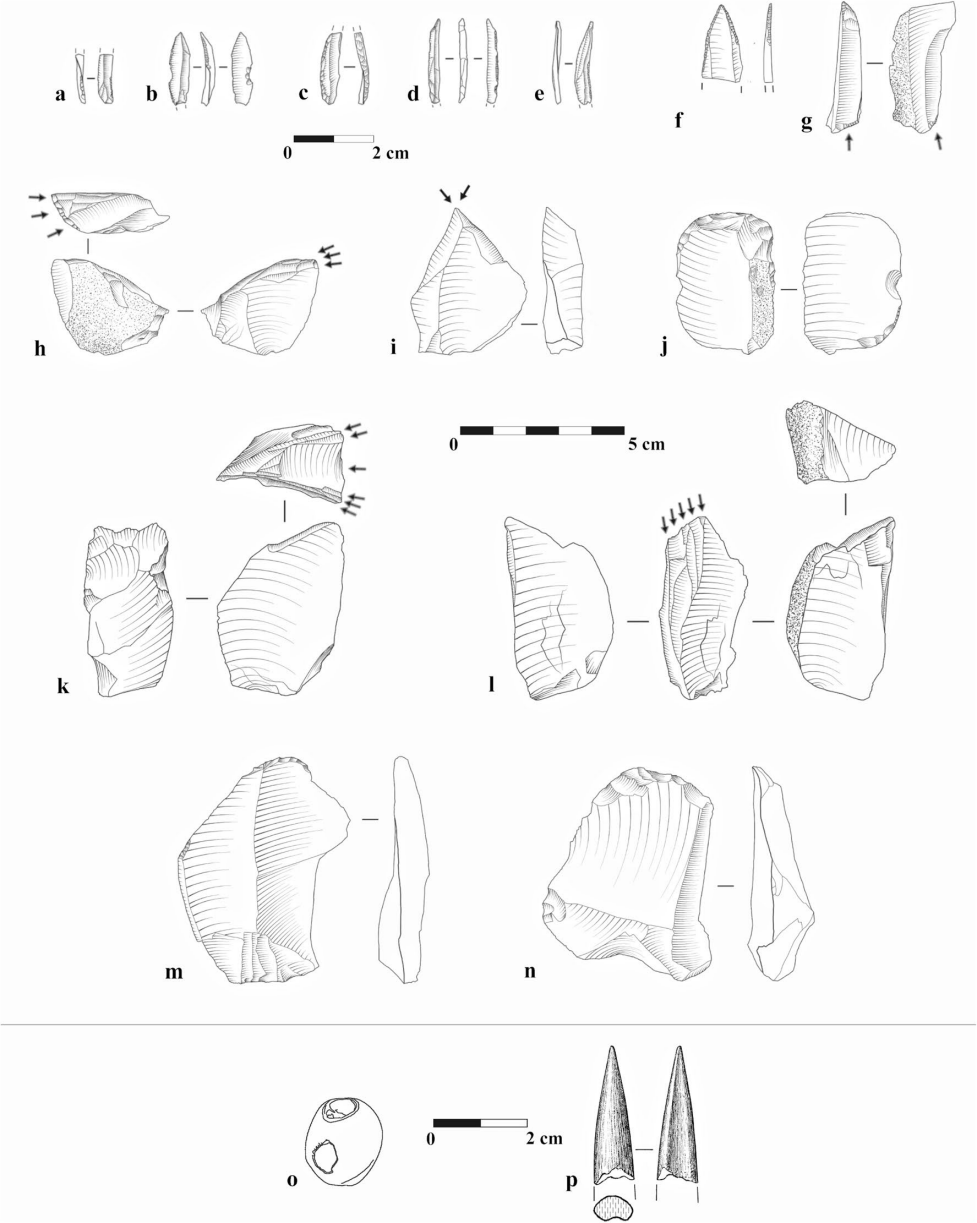
Typical components of the Arkov-Divshon assemblages from Nahal Rahaf 2 Rockshelter: **a**, **e** bladelets with partial, fine retouch; **b** retouched bladelet with an inversed notch; **c** bladelet with back curved by abrupt retouch; **d** bladelet with inverse retouch; **f** el-Wad point (distal fragment); **g** burin on a natural plane; **h**, **k**–**l**) lateral carination (carinated burins); **i** dihedral burin; **j**, **m**–**n** endscrapers; **o** perforated marine shell (*Tritia gibbosula*); **p** bone awl

The study of the lithic assemblages from NR2 indicated an industry that was equally focused on the production and use of thick flakes and blades on the one hand and twisted bladelets on the other (Shemer et al., 2023). Several refitted sequences implied the prevalence of a two-stage reduction sequence, where stage 1 was used to produce thick artifacts, and in stage 2, twisted bladelets were produced using lateral carination, applied on items reduced in stage 1 (Fig. 2). Among the tools, the microlithic component is dominant, averaging ca. 42% of the retouched tools category. Bladelets most commonly presented partial, light retouch on one or both lateral edges (Fig. 3a–e). Bladelets with alternate retouch (i.e., Dufour bladelets) were present but not common, as were el-Wad points (Fig. 3f; Shemer et al., 2023). Macrolithic tools were most commonly shaped from flakes, with a notable preference for thick items, (i.e., thicker than 1 cm), commonly produced in stage 1. Endscrapers are the most common tool-type, specifically flat endscrapers (Fig. 3m–n), followed by burins, mostly of the dihedral type (Fig. 3i). Burins on truncation are rare (Shemer et al., 2023).

Most intriguing was the presence of several bone awls (Fig. 3p), previously not considered integral components of Arkov-Divshon assemblages, and of perforated marine shells (Fig. 3o), indicating contact with the Mediterranean region.

Based on a comprehensive characterization of many of the sites associated with the Arkov-Divshon, conducted by J. Williams (2003), the flint industry reflected in NR2 was suggested to bear the closest resemblance to the sites of Arkov (D22; Marks & Ferring, 1976), Ein Aqev (D31; Williams, 2003), Ramat Matred I (Gilead, 1993), and possibly also Har Horsha I (Belfer-Cohen & Goring-Morris, 1986) and Palm View 1: areas 3–4 (Parow-Souchon, 2020; Schyle, 2015; Schyle & Richter, 2015). The association relied on both the reconstruction of a primary, two-stage reduction sequence and on the abundance of lateral carination, while other aspects, such as the capacity of the microlithic component, presented high variability (Shemer et al., 2023). Notably, the presence of perforated marine shells was also reported from the Arkov-Divshon site of Ein-Aqev (Marks, 1976b; Williams, 2003).

Fifteen samples dated independently in two laboratories provided a chronological range of 39.5–34.0 ky cal BP. A more confined range of 37.5–34.0 ky cal BP was suggested based on the stratigraphy and the contextual quality of the samples (Shemer et al., 2023). Considering the current chrono-cultural models of the Levantine Upper Paleolithic that places the Arkov-Divshon in the LUP, these results were unexpected.

### Manot Cave, the Levantine Aurignacian, and the Atlitian of the Mediterranean Region

The second case study, Manot Cave, is located in the western Galilee, ca. 9 km east of the Mediterranean shoreline. The entrance to the cave collapsed ca. 30 kya, contributing to the creation of a stable, humid environment within the cave, which resulted in the good preservation of organic remains. The cave is composed of a main, elongated hall (ca. 100 × 30 m) that branches into two smaller chambers. The primary area of human activity was identified near the entrance to the cave, on a plateau located in the topographically most elevated part of the cave. From there, a large talus steeply slopes to the southwest, ending in a second plateau (e.g., Barzilai et al., 2016, 2021; Marder et al., 2021). Excavations in the cave implied intensive human and carnivore activity at the site and its vicinity roughly ca. 55–33 kya (e.g., Abulafia et al., 2021; Alex et al., 2017; Barzilai et al., 2016, 2021; Hershkovitz et al., 2015; Marder et al., 2017, 2018, 2021; Shemer et al., 2024). The main occupations were ascribed to the Upper Paleolithic period, specifically to the Early Ahmarian, Levantine Aurignacian, and Atlitian (previously referred to as “post-Levantine Aurignacian”; Shemer et al., 2024).

In the entrance hall, ca. 2.5 m of archaeological accumulation contained three distinct in situ occupation phases. Characterization of the flint industries alongside a high-resolution dating enabled the establishment of a refined definition and chronology for each of the cultural entities (Shemer et al., 2024).

The oldest, Phase 3, currently including unaffiliated lithic industries, was defined at the base of the sequence and will not be discussed here (Shemer et al., 2024).

**Fig. 4:**
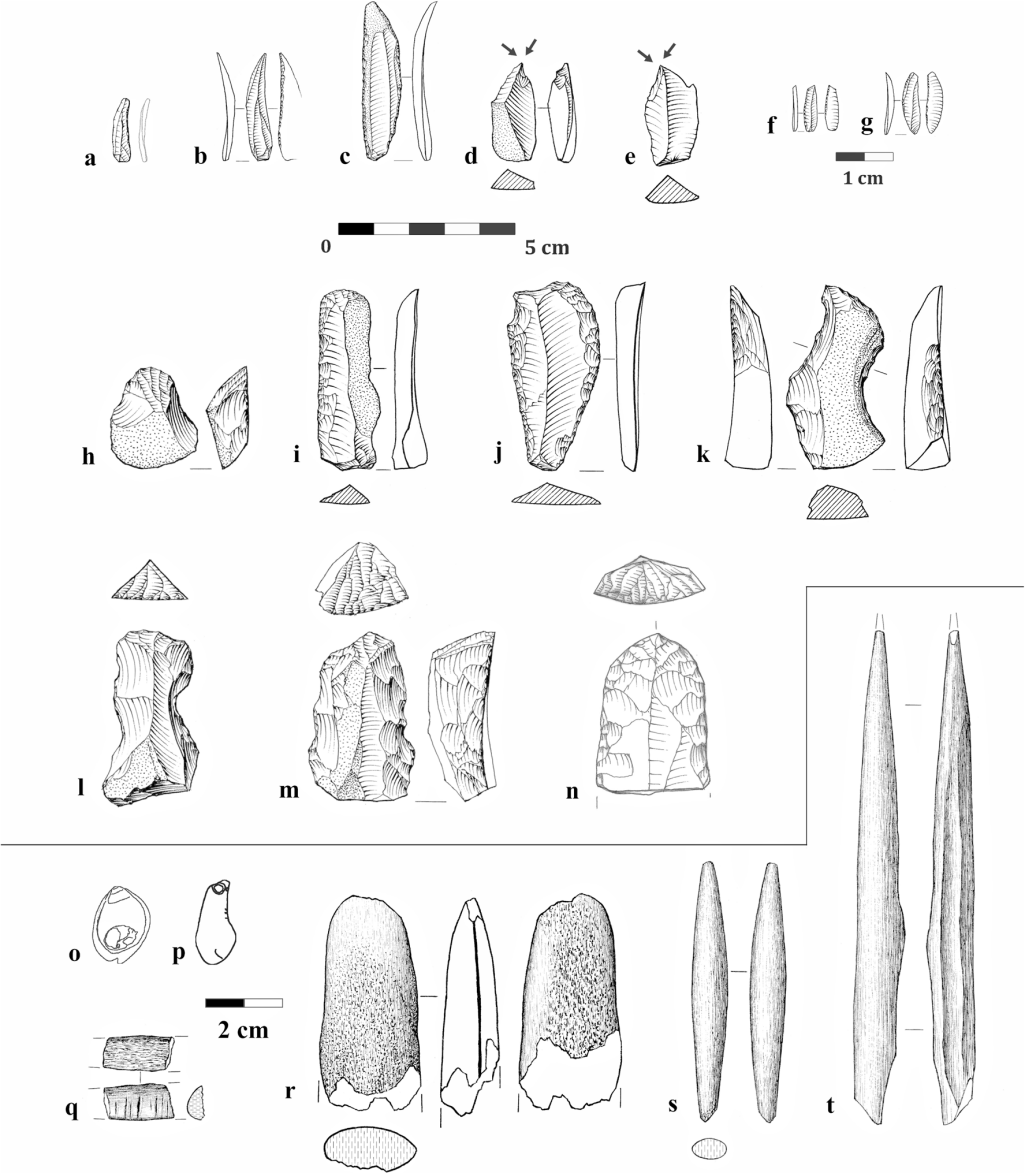
Typical components of the Levantine Aurignacian assemblages from Manot Cave (Phase 2): **a** bladelet with partial fine retouch; **b** bladelet with inverse retouch; **c** blade with complete, bilateral retouch; **d**–**e** dihedral burins; **f**–**g** Dufour bladelets, shaped on products of frontal carination; **h** frontal carination (carinated endscraper); **i** endscraper and Aurignacian retouch; **j** blade with bilateral Aurignacian retouch; **k** blade with two wide notches; **l** frontal carination on a strangled blade (atypical); **m**–**n** frontal carination and bilateral Aurignacian retouch; **o** perforated marine shell (*Nassarius* spp.); **p** polished-tooth pendant; **q** fragment of incised scapula; **r** antler intermediate piece; **s** antler projectile point; **t** bone awl

Phase 2 was attributed to the Levantine Aurignacian (Shemer et al., 2024; Layers VI–IV and probably also Layers VII–VIII that were excluded from that study) and was characterized by a diversified lithic industry, where distinct trajectories were used for the reduction of different target products: blades, flakes, curved/twisted bladelets, and straight bladelets. The technological breakdown of the analyzed assemblages suggested that blade production was primarily conducted off-site (Shemer et al., 2024). These blades were an integral component of the Levantine Aurignacian industries, used for shaping typological hallmarks such as scrapers and blades with invasive, scalar retouch (i.e., “Aurignacian retouch”; Fig. 4i, j, m–n), but also items with only marginal or limited retouch included in the general “retouched blades” category. Flakes were produced in a separate sequence, often from multiple-platform cores. These were often used for the shaping of frontal carination (i.e., carinated endscrapers; Fig. 4h, l–n). Notably, the curved bladelets produced from the shaping of frontal carination were exclusively used to shape Dufour bladelets (Fig. 4f–g). Finally, straight bladelets were produced separately from designated bladelet cores and constitute the majority of microlithic tools (Fig. 4a–b). The occasional use of Middle Paleolithic artifacts via recycling marks an additional Levantine Aurignacian attribute found in Manot Cave, Phase 2 (Belfer-Cohen & Bar-Yosef, 2015; Marder et al., 2018; Shemer et al., 2024).

Additional integral components of the Levantine Aurignacian assemblages are rich osseous industries and personal ornaments, including incised scapulae, polished-tooth pendants, perforated marine shells, bone awls, and antler projectile points (Fig. 4o–t; Tejero et al., 2016, 2021). The Levantine Aurignacian occupation at Manot Cave was radiocarbon dated to 37.5–36.0 ky cal BP (Shemer et al., 2024).

Based on the techno-typological attributes of the lithic assemblages, and the presence of the highly diagnostic osseous artifacts, Manot Cave, Phase 2 is comparable to the Levantine Aurignacian assemblages from Ksâr ‘Akil Rockshelter Phase 5 (Williams & Bergman, 2010; Phase VI in Bergman et al., 2017; M.S., personal observations); Hayonim Cave Layer D (Belfer-Cohen & Bar-Yosef, 1981); Sefunim Cave Level V (Shimelmitz et al., 2018); Kebara Cave Layers II–I (Bar-Yosef & Belfer-Cohen, 2019); Raqefet Cave Layers IV–II (Lengyel, 2007); and el-Quseir Rockshelter Layer C (Perrot, 1955) as was suggested by Shemer et al. (2024).

**Fig. 5:**
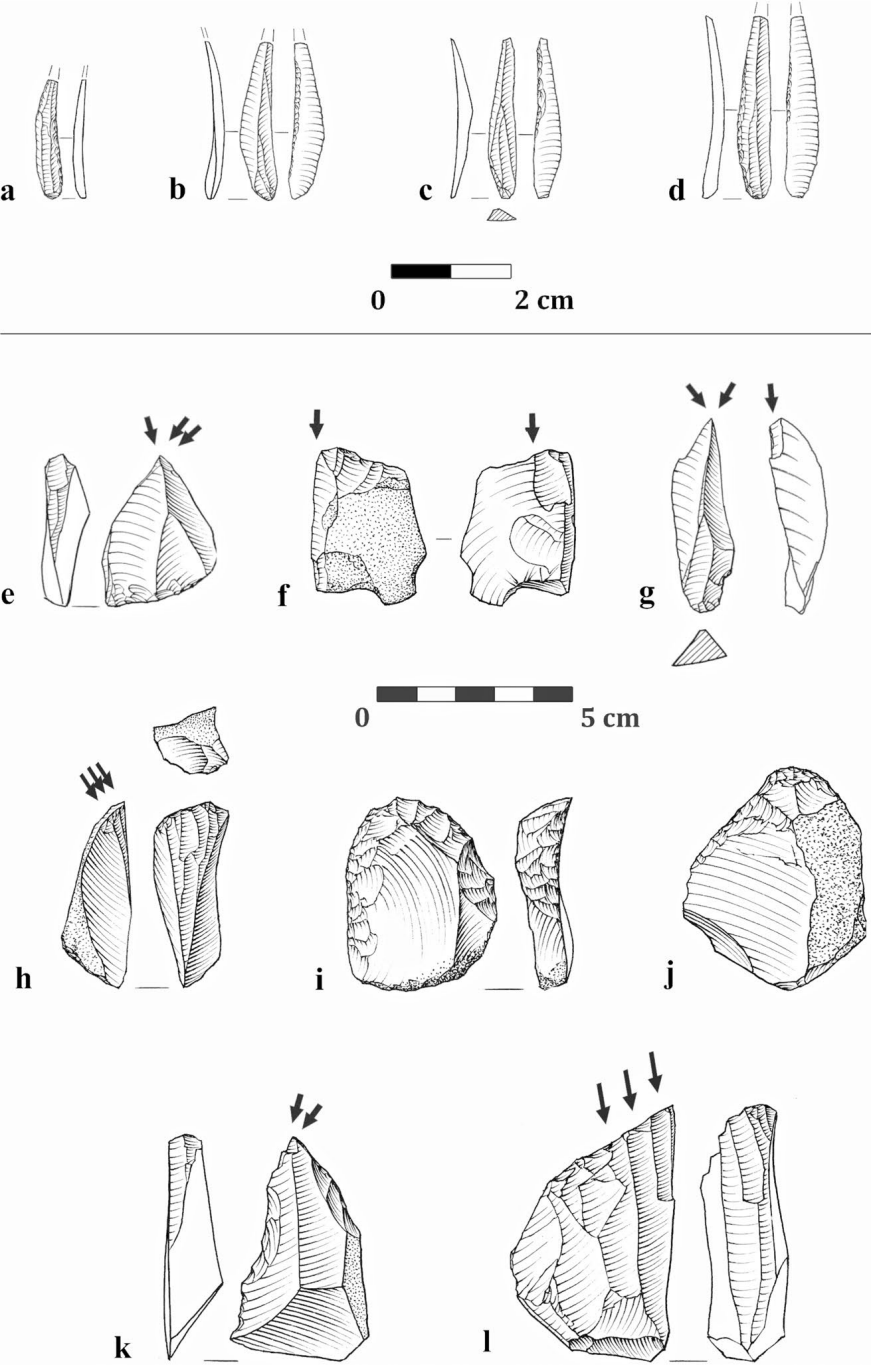
Typical components of the Atlitian lithic industries from Manot Cave (Phase 1): **a** bladelet with complete retouch; **b** bladelet with inverse retouch; **c**–**d** Dufour bladelets; **e** multiple dihedral burin; **f** burin on a concave truncation; **g** dihedral burin; **h**, **l** lateral carination; **i**–**j** endscrapers on flakes; **k** burin on an oblique truncation

Manot Phase 1, defined as Atlitian (Shemer et al., 2024; Layers III–I—previously referred to as “post-Levantine Aurignacian,” e.g., Barzilai et al., 2016; Marder et al., 2021), is characterized by reduced technological variability and increased dominance of the microlithic component. Two primary reduction methods are evident: The first is a focused production of thick flakes and blades (i.e., thicker than 1 cm) that were subsequently used to produce twisted blades and bladelets via lateral carination (i.e., carinated burins). These twisted bladelets constitute the majority of the microlithic component. A second reduction sequence was used to produce bladelets from single-platform cores. These cores are distinguished from the bladelet cores of Phase 2 by their extremely narrow reduction surface (ca. 1 cm wide; Shemer et al., 2024). Among the tools, the microlithic component (Fig. 5a–d) constitutes an average of ca. 40%. Burins are the most abundant among macrolithic tools, comprising ca. 13–21% in the Atlitian layers. Thirty to fifty percent of them were shaped on truncation, notch, or lateral preparation varieties (Fig. 5f, k); Shemer et al., 2024). Notably, an increase is observed in the dominance of the microlithic component among the tools and of burins on truncation among the burins between the oldest Atlitian Layer III and the youngest Layer I (Shemer et al., 2024). Based on the technological reconstruction of a prominent two-stage reduction sequence, the primary contribution of lateral carination to bladelet production, and the abundance of burins on truncation among the burins (≥ 30%), Manot Phase 1 was suggested to correspond to the Atlitian assemblages of Ksâr ‘Akil Rockshelter, Phase 6 (Williams & Bergman, 2010; Phase IV in Bergman et al., 2017; M.S. personal observations) and Yabrud II Rockshelter, Layer 1 (Ghazi, 2013; Ziffer, 1981). Techno-typological similarities are also suggested with el-Wad Cave, Layer C (Garrod & Bate, 1937), and el-Khiam Terrace, Layer E (Neuville, 1951), albeit the available data for these assemblages is severely meager.

Radiocarbon dating of Manot Phase 1 provided an age range of ca. 34.5–33.1 ky cal BP (Alex et al., 2017; Shemer et al., 2024). These are minimum ages, as all dated samples were collected from the youngest Layer I, while absolute dating was not achieved for the two underlying Atlitian layers (Layers II–III). However, when considering the absolute chronology of the Atlitian Phase 6 in Ksâr ‘Akil Rockshelter (Bosch et al., 2015; Douka et al., 2013) alongside the minimum ages of the underlying Phase 2 in Manot Cave, a general age range of ca. 35.0–33.0 ky cal BP can be suggested for the Atlitian (Shemer et al., 2024).

## Revisiting the Issue: The Levantine Upper Paleolithic “Flake-Based” industries and Their Relation to the Levantine Aurignacian

Comprehensive analyses of the flint assemblages from Manot Cave and Nahal Rahaf 2 Rockshelter (Shemer et al., 2023, 2024) provided the techno-typological basis for testing possible connections—developmental or adaptive—between the Levantine Aurignacian and the “flake-based” industries, the Arkov-Divshon and the Atlitian. In search of shared traditions, approaches, or preferences that may be used to extrapolate on the possible assimilation between these cultural entities, an emphasis was placed on the technological attributes of the lithic industries, as they are slower to change, less affected by site function, and considered to reflect deeper, more inherent connections, compared to typological attributes (e.g., Bar-Yosef & Belfer-Cohen, 2013; Kadowaki, 2013; Pelegrin, 1990, 1991, 2000). Nevertheless, typological aspects of the assemblages, as well as other features such as the presence and attributes of osseous industries and personal ornaments, were also examined, being integral elements of cultural characterization.

The first indicator that a revision of the accepted chrono-cultural model is required was the dating results of the Arkov-Divshon presence in NR2. Taken at face value, the suggested age range of ca. 37.5–34.0 ky cal BP places the Arkov-Divshon within the EUP rather than the LUP, implying its co-inhabitance of the southern Levant alongside the Levantine Aurignacian and possibly with the Early Ahmarian. This chronological overlap hinders a scenario encompassing its local evolvement from the Levantine Aurignacian.

Intriguingly, comparative analyses of the flint industries indicate a close resemblance between the Arkov-Divshon and the Atlitian, whereas the Levantine Aurignacian seems to encompass distinctly different technological and typological attributes. The observed resemblance between the former two is reflected primarily in the technological aspects: the Arkov-Divshon and the Atlitian share a similar reduction approach in their reliance on a primary two-stage sequence and in the systematic use of lateral carination for bladelet production (e.g., Shemer et al., 2023, 2024; Williams, 2003). Accordingly, the flint industry in both cultural entities equally emphasized the production of thick flakes and blades on the one hand and twisted bladelets on the other (e.g., Shemer et al., 2023, 2024; Williams, 2003).

The primary difference between the Atlitian and the Arkov-Divshon flint industries is typological, marked by the abundance of burins on truncation among the Atlitian tool assemblages, whereas they are less common in some Arkov-Divshon assemblages, as shown in the case study of NR2 (Shemer et al., 2023). Nevertheless, the presence of burins on truncation was reported in the Arkov-Divshon sites of Har Horesha I, Arkov (D22), Ein Aqev (D31), and Palm View 1, albeit not in equal dominance to that reported from Atlitian assemblages (Table 1; Belfer-Cohen & Goring-Morris, 1986; Marks, 1976b; Parow-Souchon, 2020; Schyle, 2015; Williams, 2003).

Other differences between the Arkov-Divshon and the Atlitian flint industries seem to fall within the variability range of each cultural entity. For example, the general dominance of burins among the tools was often associated with the latter (e.g., Belfer-Cohen et al., 2004; Ghazi, 2013; Shemer et al., 2024; Williams, 2003), despite the predominance of endscrapers described in the assemblages from el-Wad Cave, Layer C, and el-Khiam Terrace, Layer E (Garrod & Bate, 1937; González Echegaray, 1964; Neuville, 1951). For the Arkov-Divshon, the dominance of endscrapers vs. burins was noted in NR2 (Shemer et al., 2023), while in other Arkov-Divshon assemblages, it was burins that outnumbered the endscrapers (e.g., Belfer-Cohen & Goring-Morris, 1986; Gilead, 1993; Williams, 2003).

The similarities between the Arkov-Divshon and the Atlitian flint industries are inherent. Hallmark attributes, such as the abundance of lateral carination commonly associated with the former and burins on truncation associated with the latter, were found in this study to be integral components in the flint assemblages of both cultural entities. In addition, the assemblages from the two case studies presented here indicate an increasing dominance of the microlithic component, possibly reflecting a general process of microlithization (Shemer et al., 2023, 2024). The technological and typological affinities obscure the distinction between the Arkov-Divshon and the Atlitian and indicate shared lithic traditions, supporting an observation made by J. Williams two decades ago (Williams, 2003). Based on the shared reduction approach mentioned above, Williams grouped assemblages from the two cultural entities, suggesting that the geographical distribution of the sites might reflect a migration route from the Mediterranean to the arid regions of the southern Levant (Williams, 2003). Considering the chronological frames now available, it is plausible that the Atlitian represents a younger manifestation of the Arkov-Divshon, possibly indicating population movements from arid environments to the Mediterranean woodland region. 

Compared to the Arkov-Divshon and the Atlitian, the Levantine Aurignacian flint industries display a wider technological diversity. Distinct reduction sequences were used for the production of different target artifacts: large blades, possibly produced off-site; flakes from single-platform and multiple-platform cores; straight bladelets from designated cores; and small, curved bladelets—products of frontal carination. Blades were highly favored for retouch in Levantine Aurignacian industries (Table 1), and the bladelet component was present but not dominant, constituting ca. 20% or less of both the debitage and tools categories (Table 1, e.g., Belfer-Cohen & Bar-Yosef, 1981; Lengyel, 2007; Shemer et al., 2024; Williams, 2003; Williams & Bergman, 2010).

Frontal carination, a hallmark of Levantine Aurignacian industries, was secondary in the Atlitian and the Arkov-Divshon, where lateral carination was more common. The comparison between the industries further suggests that these two types of carination served different purposes: frontal carination of the Levantine Aurignacian (Phase 2) in Manot Cave was often accompanied by other forms of retouch, implying its use for multiple functions (Shemer et al., 2024). Reduction surface attributes and the scarcity of typical maintenance spalls indicated few rounds of reduction. Thus, there is no clear evidence for systematic bladelet production from frontal carination despite the targeted use of frontal-carination-bladelets for the shaping of Dufour bladelets. These were too few to define a designated, systematic production rather than the use of reduction by-products (Shemer et al., 2024). In contrast, lateral carination in the Atlitian and the Arkov-Divshon flint industries was an integral component of the two-stage reduction sequence and a primary contributor to the production of bladelets. Intriguingly, in the Atlitian assemblages of the Jordan Valley, where lateral carination is scarce, the abundance of the microlithic component is also notably low (Belfer-Cohen et al., 2004; Goring-Morris, 1980).

Extending the comparison, additional distinctions arise as the Arkov-Divshon and Atlitian assemblages in both case studies lack key elements of the Levantine Aurignacian. For example, Aurignacian retouch is absent in both the Arkov-Divshon and Atlitian assemblages, and aside from the marked presence of bone awls, there is no indication of the presence of complex osseous industries (e.g., Belfer-Cohen & Bar-Yosef, 1981; Goring-Morris, 1980; Lengyel, 2007; Shemer et al., 2023, 2024; Shimelmitz et al., 2018; Williams, 2003; Williams & Bergman, 2010). While antler-made projectiles and intermediate pieces, polished-tooth pendants, and incised scapulae are integral components of Levantine Aurignacian assemblages, neither the finished artifacts nor production spalls were associated with either the Atlitian or the Arkov-Divshon (e.g., Shemer et al., 2023, 2024; Tejero et al., 2016, 2021).

The techno-typological differences between the Arkov-Divshon and the Atlitian on the one hand and the Levantine Aurignacian on the other might have been suggested to represent evolvement into a more time-efficient, microlithic-oriented approach, giving a time-lapse between these industries. However, considering the chronological overlap now suggested between the Levantine Aurignacian and the Arkov-Divshon, the observed differences seem too vast to be considered as two manifestations of a single tradition. Extrapolating from the archaeological evidence, two distinct cultural groups seem more likely to be reflected here: one corresponding with the Levantine Aurignacian and the other with the Arkov-Divshon and Atlitian.

## The Origins of the Levantine Aurignacian

Numerous studies have discussed the possible origins of the Aurignacian culture, trying to trace its emergence and dispersal throughout Eurasia and often equating the patterns with the arrival and establishment of modern human populations (e.g., Anderson et al., 2018; Barshay-Szmidt et al., 2018a, 2018b; Belfer-Cohen & Goring-Morris, 2014; Bon, 2002; Bordes, 2006; Conard & Bolus, 2008; Falcucci et al., 2020; Garrod, 1953; Higham et al., 2011; Mellars, 2006; Michel, 2010, 2012; Nigst et al., 2014; Tejero et al., 2021; Teyssandier & Zilhão, 2018; Teyssandier et al., 2010; Zilhão & d'Errico, 2006). From a local perspective, the study presented here stresses the already established view of the Levantine Aurignacian as representing traditions and ideas that were foreign to the cultural entities of the southern Levant. The techno-typological characteristics presented in this study suggest a low probability of evolutionary connections with the Arkov-Divshon and/or the Atlitian, further highlighting the detachment of the Levantine Aurignacian assemblages from the regional record. With the short chronology suggested for the Levantine Aurignacian (Phase 2) in Manot Cave, the archaeological record seems to show increasing support for a scenario in which a foreign band of hunter-gatherers arrived in the southern Levant ca. 38 ky cal BP, bringing with them traditions and ideas that disappeared a few millennia later.

 Searching for the roots of the Levantine Aurignacian within the Aurignacian technocomplex of Eurasia is intriguing, as Levantine assemblages contain a distinct set of attributes that is comparable to both the Early and Late phases of the Aurignacian of western Europe (Archaic/Aurignacian I and Evolved/Aurignacian II, respectively, e.g., Bon, 2002; Bordes, 2006; Davies, 2001; Dinnis et al., 2019; Douka et al., 2011; Tejero et al., 2016, 2021).

The west European Early Aurignacian, roughly dated ca. 43.5/40–38 ky cal BP (e.g., Barshay-Szmidt et al., 2018b; Dinnis et al., 2019; Douka et al., 2011; Higham et al., 2011; Michel, 2010; Nigst et al., 2014; Teyssandier & Zilhão, 2018), is characterized by the dominance of blade tools, including an abundance of Aurignacian retouch and “strangled” blades. The lithic industry of this phase is repeatedly described as targeted for the production of large blades and curved bladelets in two distinct sequences, where bladelets were systematically reduced from carinated cores (i.e., frontal carination with wide bladelet reduction surface, e.g., Barshay-Szmidt et al., 2018b; Bon, 2002; Bordes, 2006; Breuil, 1913; Douka et al., 2011; Michel, 2010; Teyssandier et al., 2010). Notably, split-base antler points that were regarded as highly diagnostic Early Aurignacian hallmarks in the twentieth and early twenty-first centuries have been shown in the past two decades to be confined mainly to the European Aquitaine region and, therefore, are no longer considered reliable chrono-cultural markers in other regions (e.g., Dinnis et al., 2019; Liolios, 1999, 2006).

In the Late Aurignacian, dated ca. 38–34/33 ky cal BP (e.g., Barshay-Szmidt et al., 2018b; Dinnis et al., 2019; Higham et al., 2011; Michel, 2010), the dominance of blade tools decreased, and Aurignacian retouch and “strangled” blades became uncommon. Affiliated flint assemblages indicate increased dominance of the bladelet component and targeted production of twisted bladelets from lateral carination. Increased frequencies of Dufour bladelets are also noted; these were often shaped from small bladelets, products of frontal carinated items whose bladelet reduction surfaces were purposefully narrowed (i.e., nosed and shouldered endscrapers, e.g., Anderson et al., 2018; Barshay-Szmidt et al., 2018b; Bordes, 2006; Chiotti et al., 2015; Douka et al., 2011; Michel, 2010, 2012).

The case study of Manot Cave, the Levantine Aurignacian (Phase 2), seems to present mixed attributes. The dominance of blade tools, and the abundance of Aurignacian retouch are strongly associated with the Early Aurignacian. However, in contrast to European assemblages, single-platform cores were the primary contributors to bladelet production in Manot Cave, whereas frontal carination with a wide bladelet reduction surface (e.g., Fig. 4h) is uncommon. Instead, frontal carination in Manot Cave often presents relatively narrow surfaces that would have produced small (≤ 1 cm) bladelets (e.g., Fig. 4l–n; Shemer et al., 2024). The targeted use of these bladelets for the shaping of Dufour bladelets was clearly demonstrated (Shemer et al., 2024)—an attribute associated mostly with Late Aurignacian assemblages in Europe (e.g., Anderson et al., 2018; Barshay-Szmidt et al., 2018b; Bordes, 2006; Chiotti et al., 2015; Douka et al., 2011; Michel, 2010, 2012). Nevertheless, the dominance of burins and lateral carination that is commonly associated with the European Late Aurignacian is not reflected in Levantine Aurignacian assemblages (e.g., Bar-Yosef & Belfer-Cohen, 2019; Belfer-Cohen & Bar-Yosef, 1981; Garrod & Bate, 1937; Lengyel, 2007; Marder et al., 2021; Neuville, 1951; Perrot, 1955; Shimelmitz et al., 2018).

Chronologically, the ages received for the Levantine Aurignacian are young compared to the west European Early Aurignacian occupations. The current state of research suggests an age range of ca. 38–34/33 ky cal BP for the Levantine Aurignacian (Alex et al., 2017; Bosch et al., 2015; Douka et al., 2013; Lengyel, 2007; Lengyel et al., 2006), correlating perfectly with the ages of the Late Aurignacian in Europe. The shorter chronology of ca. 37.5–36.0 ky cal BP, recently suggested based on the Manot Cave sequence, places the Levantine Aurignacian closer to the latest noted appearance of the west European Early Aurignacian, but nonetheless within the European Late Aurignacian chronology.

Intriguingly, a somewhat similar admixture of attributes has been reported in recent years from a few sites in western Europe, such as Layer 8 in Abri Pataud (Michel, 2010, 2012), the open-air site of Régismont-le-Haut (e.g., Anderson et al., 2018; Barshay-Szmidt et al., 2018b), and Unit 2 in Les Cottés (Jacobs et al., 2015; Roussel & Soressi, 2013), all dated roughly ca. 38–36 ky cal BP. The prevalence of Early Aurignacian attributes alongside the absence of Late Aurignacian hallmarks in the form of lateral carination and burin dominance on the one hand and the relatively young chronology on the other hand led to the suggestion that these assemblages reflect a late segment within the Early Aurignacian or an intermediate phase between the Early and Late Aurignacian industries (i.e., “Middle Aurignacian”; Anderson et al., 2018; Barshay-Szmidt et al., 2018b; Jacobs et al., 2015; Michel, 2010, 2012).

Within the framework of the present research, a similar suggestion might be made for the Levantine Aurignacian. The techno-typological attributes of Levantine assemblages correlate better to the Early rather than the Late phase of the west European Aurignacian technocomplex. Considering their chronology, the Levantine Aurignacian is likely to reflect population movement at the peak of expansion of Early Aurignacian traditions throughout Eurasia, shortly before their replacement by Late Aurignacian industries.

## Cultural Dynamics and Migration Patterns: Revising the Chrono-cultural Model of the Levantine Upper Paleolithic

The study presented here provides a new perspective on the Early Upper Paleolithic period in the southern Levant. Technological and typological analyses and radiocarbon chronology established the distinction of the “flake-based industries” from the Levantine Aurignacian on the one hand, stressing the foreign nature of the latter and its association with incoming populations. On the other hand, inherent similarities were identified between the Arkov-Divshon and the Atlitian, suggesting a shared lithic tradition for the two entities, implying that both reflect a single cultural group. Adding these observations to the bank of data accumulated throughout almost a century of research, three main cultural entities can now be securely associated with the Early Upper Paleolithic period in the southern Levant: the Ahmarian, the Levantine Aurignacian, and the Arkov-Divshon/Atlitian. Each contains a distinct set of technological and typological attributes that may imply different sets of social behaviors (Table 2).

**Table 2:**
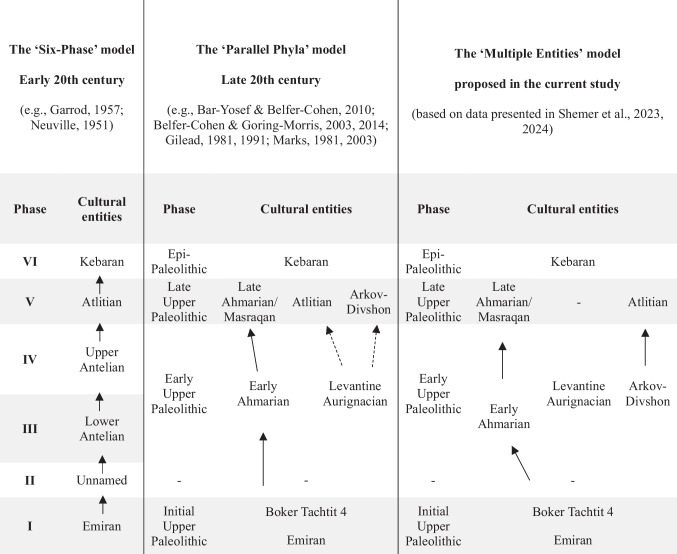
Primary steps in the evolvement of the chrono-cultural model of the Levantine Upper Paleolithic, including the adjustments suggested in the current study. Arrows mark suggested techno-typological continuity. Dashed arrows marked tentatively suggested continuity, which have not been fully established

A significant contribution to the study of the Levantine Upper Paleolithic is the absolute chronology achieved for the Arkov-Divshon at NR2 Rockshelter, implying the antiquity of a cultural entity commonly regarded as a much younger manifestation (Shemer et al., 2023). Thus, further supporting the observations gained by the lithic industries: not two, but at least three cultural entities co-resided in the southern Levant ca. 40–30 ky cal BP. The social complexity implied by the presence of these hunter-gatherer groups in a shared geographic region raises questions regarding inter-group dynamics and the nature of potential interactions. However, it is hard to extrapolate on these subjects based solely on the archaeological record, where the resolution is rarely sufficient for identifying and characterizing daily activities and short-term processes. Nevertheless, important data is provided by the stratigraphic record of the Levantine sites, eliciting insights about geographic distribution and relative chronology.

One of the main issues in the study of cultural dynamics is the question of the nature of interaction between groups. For example, what happens to indigenous groups when a new population arrives in an already populated region? Such a scenario is suggested, for example, for the Levantine Aurignacian. Regional absolute chronology indicates the presence of Early Ahmarian populations in the Mediterranean region roughly at, or close to, the time of the arrival of Levantine Aurignacian populations (e.g., Alex et al., 2017; Bar-Yosef et al., 1996; Bosch et al., 2015; Douka et al., 2013; Kuhn et al., 2009; Rebollo et al., 2011). The archaeological record suggests some degree of replacement rather than the co-existence of the two, an observation based primarily on the inner stratigraphy of the sites. Thus, when present together in a single sequence, Levantine Aurignacian layers always overlay the ones ascribed to the Early Ahmarian (e.g., Abulafia et al., 2021; Bar-Yosef & Belfer-Cohen, 2019; Barzilai et al., 2016; Garrod & Bate, 1937; Marder et al., 2017; Neuville, 1951; Perrot, 1955; Williams & Bergman, 2010). None of the sites display intermittent occupations that would correspond with the co-existence of the two cultural entities in the vicinity of a specific site. Nevertheless, the possibility that “pockets” of Early Ahmarian groups remained in the Mediterranean region throughout the Levantine Aurignacian occupation cannot be discarded. Similarly, when present in a single sequence, Atlitian layers always overlay the ones associated with the Levantine Aurignacian (e.g., Garrod & Bate, 1937; González Echegaray, 1964; Marder et al., 2021; Neuville, 1951; Shemer et al., 2024; Williams & Bergman, 2010), likewise implying a replacement of the Levantine Aurignacian by Arkov-Divshon/Atlitian populations ca. 35 ky cal BP.

While the stratigraphic records provide some indication regarding the Mediterranean region, there is much more ambiguity regarding processes that occurred at the same time in the arid regions of the Levant. There, many of the sites are open-air and single-layered, often showing signs of deflation and post-depositional erosion (e.g., Ferring, 1976; Mark & Ferring, 1976; Marks, 1977b; Baruch & Bar-Yosef, 1986; Belfer-Cohen & Goring-Morris, 1986; Gilead, 1993; Gilead & Bar-Yosef, 1993; Schyle & Richter, 2015; Parow-Souchon, 2020). Other sites present a multi-layered sequence but were ascribed in their entirety to a single cultural entity—the Arkov-Divshon, further impeding stratigraphy-based relative chronology (Marks, 1976b; Shemer et al., 2023). Therefore, the degree of interaction and possible contemporaneity between the Arkov-Divshon and the Ahmarian industries in these regions are indeterminable. Similar difficulties arise in the attempt to estimate the chronological correspondence of the Arkov-Divshon in the Negev Desert, Sinai Peninsula, and southern Jordan, on the one hand, and the dated Arkov-Divshon assemblages of the Judean Desert and the Atlitian of the Mediterranean region, on the other hand.

**Fig. 6:**
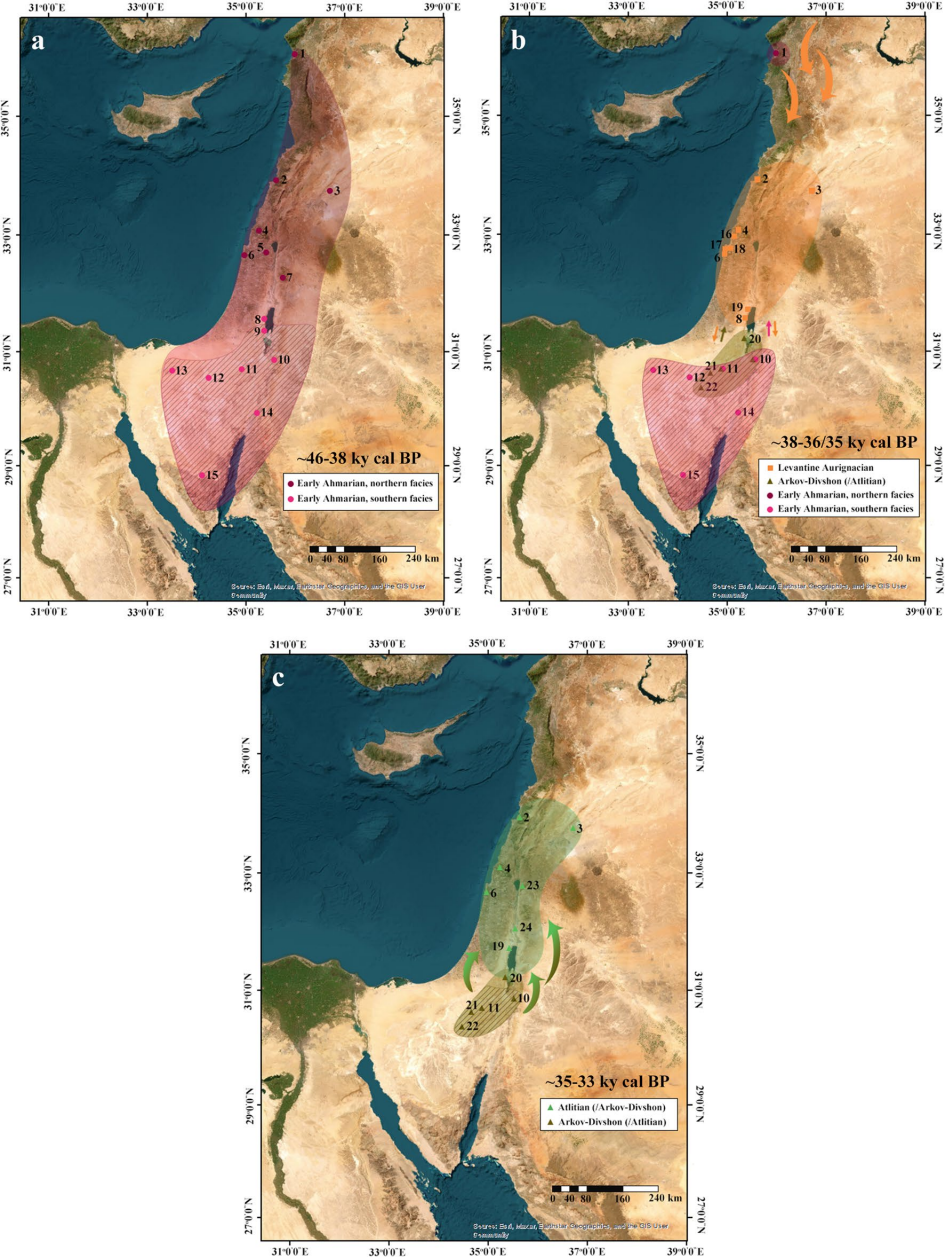
Suggested model of cultural dynamics in the Levantine Upper Paleolithic period, showing possible migration patterns ca. 46–38 ky cal BP (**a**), ca. 38–36/35 ky cal BP (**b**), and ca. 35–33 ky cal BP (**c**). Curved arrows mark primary areas and direction of migration. Small arrows represent assumed interaction between cultural entities. Diagonal lines mark areas in which the chronological data (both absolute and relative) is severely lacking. (1) Üçağızlı; (2) Ksâr ‘Akil; (3) Yabrud II; (4) Manot; (5) Qafzeh; (6) el-Wad/Kebara; (7) Mughr el-Hamamah; (8) el-Quseir; (9) Erq el Ahmar; (10) Wadi Hasa/Wadi Sabra sites; (11) Boker Tachtit/Boker sites; (12) Qadesh Barnea 601; (13) Lagama sites; (14) Jebel Qalkha sites; (15) Abu Noshra; (16) Hayonim; (17) Sefunim; (18) Raqefet; (19) el-Khiam; (20) Nahal Rahaf 2; (21) Ramat Matred sites; (22) Har Horsha I; (23) Nahal Ein Gev I; (24) Fazael IX

Nevertheless, the accumulated data allows us to suggest a partial reconstruction of migration patterns in the Levantine Upper Paleolithic period, encompassing two major shifts. The Levantine Early Upper Paleolithic seems to have seen the extensive presence of Early Ahmarian populations in the Mediterranean region (Fig. 6a). In the arid regions, the chronological data does not allow a secure determination, as most of the dated samples were obtained from open-air sites and analyzed by the end of the twentieth century, prior to the establishment of the rigorous protocols applied nowadays. Therefore, many of them present wide error margins and dated sites often present a broad scatter, falling somewhere in the range between ca. 50/45–30 ky cal BP (e.g., Boaretto et al., 2021; Gilead & Bar-Yosef, 1993; Kadowaki et al., 2015; Marks, 1977b; Phillips, 1988). Nonetheless, the stratigraphy of el-Quseir in the Judean Desert, where a sequence of Early Ahmarian and Levantine Aurignacian layers was identified, presents evidence for the presence of Ahmarian populations outside the Mediterranean region prior to the arrival of the Levantine Aurignacian (Gilead, 1981; Perrot, 1955).

The arrival of the Levantine Aurignacian ca. 38 ky cal BP marks the disappearance of Early Ahmarian populations from most key sites in the Mediterranean region and the northern Judean Desert (Fig. 6b, e.g., Abulafia et al., 2021; Bar-Yosef & Belfer-Cohen, 2019; Garrod & Bate, 1937; Marder et al., 2021; Neuville, 1951; Perrot, 1955; Williams & Bergman, 2010). It is possible that the Early Ahmarian groups were pushed at that point to the arid regions of the Levant; however, the limited chronological data available implies its disappearance from there as well, shortly after. It is possible that by that time, Early Ahmarian industries had already started placing an increasing focus on microliths, marking the beginning of their evolvement into Late Ahmarian/Masraqan industries (e.g., Kadowaki, 2013). The only indication of a possible long-term Early Ahmarian presence in the Levant after the arrival of the Levantine Aurignacian comes from the site of Üçağızlı, where no occupation layers associated with the latter were identified (Kuhn et al., 2009). The chronology of the site, based on radiocarbon dating of shell and charcoal samples, implies the possibility of Ahmarian presence there until ca. 32.5 ky cal BP (Douka et al., 2013; Kuhn et al., 2009). However, this young chronology is viewed with caution by some scholars (e.g., Alex et al., 2017).

Levantine Aurignacian populations settled primarily in caves and rockshelters within the Mediterranean region (e.g., Bar-Yosef & Belfer-Cohen, 2019; Belfer-Cohen & Bar-Yosef, 1981; Garrod & Bate, 1937; Lengyel et al., 2005; Marder et al., 2021; Shemer et al., 2024; Shimelmitz et al., 2018; Williams & Bergman, 2010; Ziffer, 1981). The archaeological record suggests higher occupational intensity compared to the Early Ahmarian, manifested in elevated find densities and increased targeting of small game and birds (Belfer-Cohen & Bar-Yosef, 1981; Rabinovich, 2003; Shemer et al., 2024; Shimelmitz et al., 2018; Yeshurun et al., 2021). A multi-phased hearth from Manot Cave further supports this claim, suggesting repeated occupations over a short period of time (Shemer et al., 2024).

In the southern Judean Desert, the presence of Arkov-Divshon populations is indicated in close proximity to the estimated arrival of the Levantine Aurignacian to the Mediterranean region (Shemer et al., 2023). Chronological data on this cultural entity is still severely lacking, and it is unclear whether the Arkov-Divshon occurrences in the Negev Desert, the Sinai Peninsula, and southern Jordan can also be ascribed to this phase. Hypothetically, these sites may mark the filling of a “void” created by the disappearance or the depleting presence of the Early Ahmarian in these regions. The finds from NR2 Rockshelter imply highly desert-adapted social behaviors, including the targeting and field butchering of small- and medium-sized ungulates and the preferred use of juniper as fuel for fire (Marom et al., 2022; Shemer et al., 2023). The degree of social interactions between the Arkov-Divshon and Levantine Aurignacian is still a research subject. The presence of perforated marine shells in Arkov-Divshon sites implies contact with the Mediterranean region (Marks, 1976b; Shemer et al., 2023), suggesting that perhaps the two cultural entities were aware of each other. The presence of Levantine Aurignacian in the site of el-Quseir, in the northern Judean Desert, further supports this suggestion, indicating geographic proximity and possible utilization of similar regional resources (e.g., water sources, game herds).

Based on absolute chronology from Ksâr ‘Akil Rockshelter and from Manot Cave, another major shift occurs ca. 36/35 ky cal BP, with the appearance of the Atlitian in these sites, replacing the Levantine Aurignacian in both (Fig. 6c; Alex et al., 2017; Bergman et al., 2017; Bosch et al., 2015; Shemer et al., 2024). The circumstances leading to the disappearance of the Levantine Aurignacian are unknown. Atlitian layers are reported from several sites in the Mediterranean region, in some cases overlying the Levantine Aurignacian (e.g., Bergman et al., 2017; Garrod & Bate, 1937; González Echegaray, 1964; Neuville, 1951; Shemer et al., 2024; Williams & Bergman, 2010; Ziffer, 1981). Nevertheless, some sites present a gap following the Levantine Aurignacian layers as they were re-occupied only during the Epipaleolithic or Neolithic (e.g., Bar-Yosef & Belfer-Cohen, 2019; Bar-Yosef et al., 1996; Belfer-Cohen & Bar-Yosef, 1981; Lengyel, 2007; Lengyel et al., 2005; Perrot, 1955). Thus, the possibility that some Levantine Aurignacian population resided in the region after the arrival of the Atlitian cannot be completely excluded; however, it is generally considered unlikely.

The high techno-typological resemblance between the Atlitian and the Arkov-Divshon has been suggested here to indicate a single cultural group. Therefore, the appearance of the Atlitian in the Mediterranean region ca. 36/35 ky cal BP may be regarded as an indication for the dispersal and establishment of these cultural groups beyond the arid regions of the southern Levant. While the chronological record is insufficient in determining whether the Negev sites were still occupied at this point, in the case of NR2 Rockshelter, this timeframe marks the end of human occupation at the site. Thus, it possibly reflects a wide trend of migration towards preferable environmental conditions.

Much remains unclear regarding this phase of the Levantine Upper Paleolithic and the transition to full-fledged Epipaleolithic industries. Both techno-typological and chronological studies are required to establish a wide scope encompassing the various manifestations of the Atlitian. One important issue, for example, is represented by the Atlitian sites of the Jordan Valley: Fazael IX and Nahal Ein Gev I (Belfer-Cohen et al., 2004; Goring-Morris, 1980). Both sites are open-air, presenting lithic industries in which lateral carination and the bladelet component are severely under-represented compared to other Atlitian assemblages (e.g., Shemer et al., 2024; Williams & Bergman, 2010), while burins on truncation are highly dominant. Nevertheless, basic technological affinity is kept in the reconstruction of one primary reduction sequence, from which flakes as well as bladelets were produced (Belfer-Cohen et al., 2004). Considering the typological dominance of burins on truncation, the focus put on flake production, and the virtual absence of Aurignacian elements, the assemblages from the Jordan Valley seem to portray a close resemblance to the Atlitian/Arkov-Divshon industries. The underrepresentation of lateral carination and of the microlithic component in these assemblages emphasizes the correlation between these elements and strengthens the notion that lateral carination was a primary contributor to bladelet production in these industries (Shemer et al., 2024; Williams, 2003). Their low frequencies in the Jordan Valley sites may indicate less focus on bladelet-related activities. Therefore, the suggestion that these assemblages represent task-specific or locally adapted occurrences (e.g., Williams, 2003) seems plausible.

Another issue that was not addressed here is the question of Early Ahmarian evolvement into the Late Ahmarian/Masraqan industries, the chronology, geography, and technological characteristics of this transition, and the mechanisms leading to it. Late Ahmarian/Masraqan sites were excluded from the model suggested here due to their young chronology: ca. 33/30–23 ky cal BP (e.g., Kadowaki, 2013; Goring Morris & Belfer-Cohen, 2017). However, the scenario of local evolvement from the Early Ahmarian suggests that the roots of these industries, possibly represented as a transitional phase, should appear in the last phase of the suggested model, ca. 36/35–33 ky cal BP.

## Concluding Remarks

The two key sites analyzed in this research, Manot Cave and Nahal Rahaf 2 Rockshelter, present new insights regarding the cultural dynamics in the Levantine Upper Paleolithic period. These insights suggest revisions to the commonly accepted chrono-cultural model and indicate more social complexity than previously thought.

The study suggests a low probability of an evolutionary connection between the Levantine Aurignacian and the “flake-based” industries of the southern Levant—the Arkov-Divshon and the Atlitian. Instead, close techno-typological similarities were observed between the two, suggesting that they represent a single cultural group. The results of this study stress the foreign nature of Levantine Aurignacian industries compared to the local Levantine record. An attempt is made to correlate between the Levantine Aurignacian and the west European Aurignacian technocomplex, suggesting an affiliation of the former with the end of the Early Aurignacian in Europe.

Techno-typological characterization combined with absolute chronology achieved for the archeological sequence from NR2 indicates the presence of the Arkov-Divshon in the region as early as ca. 37 ky cal BP, placing it within the early phase of the Levantine Upper Paleolithic, alongside the Early Ahmarian and the Levantine Aurignacian. Considering the regional data accumulated in the past century, a model of population movements and possible social interaction is suggested and discussed, encompassing two major shifts. The first is marked by the arrival of the Levantine Aurignacian to the region ca. 38 ky cal BP, replacing, to a degree, the indigenous Early Ahmarian population in the Mediterranean region. The second shift, at ca. 36/35 ky cal BP, is marked by the disappearance of the Levantine Aurignacian and the arrival of the Atlitian to the Mediterranean region, possibly reflecting the dispersal of population from the arid regions.

## Data Availability

No datasets were generated or analyzed during the current study.

## References

[CR1] Abulafia, T., Goder-Goldberger, M., Berna, F., Barzilai, O., & Marder, O. (2021). A techno typological analysis of the Ahmarian and Levantine Aurignacian assemblages from Manot Cave (Area C) and the interrelation with site formation processes. *Journal of Human Evolution,**160*, 102707. 31892422 10.1016/j.jhevol.2019.102707

[CR2] Akazawa, T., Muhesen, S., Dodo, Y., Kondo, O., Mizoguchi, Y., Abe, Y., Nishiaki, Y., Ohta, S., Oguchi, T., & Haydal, J. (1995). Neanderthal infant burial from the Dederiyeh Cave in Syria. *Paléorient,**21*, 77–86.

[CR3] Alex, B., Barzilai, O., Hershkovitz, I., Marder, O., Abulafia, T., Ayalon, A., Bar- Matthews, M., Davis, L., Bar-Yosef Mayer, D., Berna, F., Caracuta, V., Frumkin, A., Goder-Goldberger, M., Hans, M. G., Latimer, B., Lavi, R., Mintz, E., Regev, L., Tejero, J.-M., et al. (2017). Radiocarbon chronology of Manot Cave, Israel, and Upper Paleolithic dispersals. *Science Advances,**3*, e1701450. 29152566 10.1126/sciadv.1701450 PMC5687856

[CR4] Anderson, L., Lejay, M., Brugal, J. P., Costamagno, S., Heckel, C., de Araujo Igreja, M., Pradeau, J. V., Salomon, H., Sellami, F., Théry-Parisot, I., & Barshay-Szmidt, C. (2018). Insights into Aurignacian daily life and camp organization: The open-air site of Régismont-le-Haut. *Quaternary International,**498*, 69–98.

[CR5] Arensburg, B., & Tiller, A. -M. (2019). What can we learn from the Mousterian Kabara hominins? In L. Meignen & O. Bar-Yosef (Eds.), *Kebara Cave, Mt. Carmel, Israel. The middle and upper paleolithic archaeology, Part II* (pp. 285–308). Peabody Museum of Archaeology and Ethnology, Harvard University.

[CR6] Bar-Yosef, O. (1970). *The Epi-Palaeolithic cultures of Palestine*. The Hebrew University.

[CR7] Bar-Yosef, O., & Belfer-Cohen, A. (1996). Another look at the Levantine Aurignacian. In A. di Cesnola & A. Montet-White (Eds.), *The Upper Paleolithic: Colloquia, XIII International congress of Prehistoric and Protohistoric sciences* (pp. 139–150). Forli.

[CR8] Bar-Yosef, O., & Belfer-Cohen, A. (1996). Another look at the Levantine Aurignacian. In A. di Cesnola, & A. Montet-White (Eds.), *The Upper Paleolithic: Colloquia, XIII International congress of Prehistoric and Protohistoric sciences* (pp. 139–150). Forli.

[CR9] Bar-Yosef, O., & Belfer-Cohen, A. (2010). The Levantine Upper Palaeolithic and Epipalaeolithic. In E. A. A. Garcea (Ed.), *Southeastern Mediterranean Peoples between 130,000 and 10,000 Years Ago* (pp. 144–167). Oxbow.

[CR10] Bar-Yosef, O., & Belfer-Cohen, A. (2013). Following Pleistocene road signs of human dispersals across Eurasia. *Quaternary International,**285*, 30–43.

[CR11] Bar-Yosef, O., & Belfer-Cohen, A. (2019). The Upper Paleolithic industries of Kebara Cave. In L. Meignen & O. Bar-Yosef (Eds.), *Kebara Cave, Mt. Carmel, Israel. The Middle and Upper Paleolithic Archaeology, Part II* (pp. 309–401). Peabody Museum of Archaeology and Ethnology.

[CR12] Bar-Yosef, O., & Phillips, J. L. (1977). *Prehistoric investigations in Gebel Magara Sinai*. Institute of Archaeology.

[CR13] Bar-Yosef, O., Arnold, M., Mercier, N., Belfer-Cohen, A., Goldberg, P., Housley, R., Laville, H., Meignen, L., Vogel, J. C., & Vandermeersch, B. (1996). The dating of the Upper Paleolithic layers in Kebara Cave, Mt Carmel. *Journal of Archaeological Science,**23*(2), 297–306.

[CR14] Bar-Yosef, O., Meignen, L., Vandermeersch, B., Goldberg, P., & Belfer-Cohen, A. (2019). The burial of the Kebara 2 Mousterian individual. In L. Meignen & O. Bar-Yosef (Eds.), *Kebara Cave, Mt. Carmel, Israel. The Middle and Upper Paleolithic Archaeology, Part II* (pp. 275–284). Harvard University.

[CR15] Barshay-Szmidt, C., Normand, C., Flas, D., & Soulier, M. C. (2018a). Radiocarbon dating the Aurignacian sequence at Isturitz (France): Implications for the timing and development of the Protoaurignacian and Early Aurignacian in western Europe. *Journal of Archaeological Science: Reports,**17*, 809–838.

[CR16] Barshay-Szmidt, C., Anderson, L., Lejay, M., Théry-Parisot, I., Burr, G. S., Mensan, R., & Bon, F. (2018b). Out of the cave and into the light: Perspectives and challenges of radiocarbon dating an open-air Aurignacian site (Régismont-le-Haut, Mediterranean France). *Journal of Paleolithic Archaeology,**1*, 247–279.

[CR17] Baruch, U., & Bar-Yosef, O. (1986). Upper Palaeolithic assemblages from Wadi Sudr. *Western Sinai. Paléorient,**12*(2), 69–84.

[CR18] Barzilai, O. (2022). The origins and destinations of the Levantine Initial Upper Paleolithic–A view from the Negev Desert, Israel. *Acta Anthropologica Sinica,**41*, 1–12.

[CR19] Barzilai, O., Hershkovitz, I., & Marder, O. (2016). The Early Upper Paleolithic period at Manot Cave, western Galilee. *Israel. Human Evolution,**31*, 85e100. 10.1016/j.jhevol.2021.103053 34456056

[CR20] Barzilai, O., Aladjem, E., Shemer, M., Zituni, R., Greenbaum, N., Boaretto, E., & Marom, N. (2020). The early Upper Paleolithic in the south Judean Desert, Israel: Preliminary excavation results from Nahal Rahaf 2 Rockshelter. *Antiquity, 94*(27), 1–8.

[CR21] Barzilai, O., Marder, O., & Hershkovitz, I. (2021). In search of modern humans and the Early Upper Paleolithic at Manot Cave: An overview. *Journal of Human Evolution,**160*, 102965. 33714606 10.1016/j.jhevol.2021.102965

[CR22] Been, E., Hovers, E., Ekshtain, R., Malinski-Buller, A., Agha, N., Barash, A., Bar-Yosef Mayer, D. E., Benazzi, S., Hublin, J. J., Levin, L., & Greenbaum, N. (2017). The first Neanderthal remains from an open-air Middle Palaeolithic site in the Levant. *Scientific Reports,**7*(1), 2958. 28592838 10.1038/s41598-017-03025-z PMC5462778

[CR23] Belfer-Cohen, A. (1980). The Aurignacian at Hayonim Cave.

[CR24] Belfer-Cohen, A., & Bar-Yosef, O. (1981). The Aurignacian in Hayonim Cave. *Paléorient,**7*, 19–42.

[CR25] Belfer-Cohen, A., & Bar-Yosef, O. (2015). Paleolithic recycling: The example of Aurignacian artifacts from Kebara and Hayonim caves. *Quaternary International,**361*, 256–259.

[CR26] Belfer-Cohen, A., & Goring-Morris, A. N. (1986). Har Horesha I: An Upper Palaeolithic site in the central Negev highlands. *Journal of the Israel Prehistoric Society,**19*, 43*-57*.

[CR27] Belfer-Cohen, A., & Goring-Morris, A. N. (2003). Current issues in Levantine Upper Palaeolithic research. In A. N. Goring-Morris & A. Belfer-Cohen (Eds.), *More than meets the eye: Studies on Upper Palaeolithic diversity in the Near East* (pp. 1–12). Oxbow.

[CR28] Belfer-Cohen, A., & Goring-Morris, A. N. (2014). On the rebound – A Levantine view of Upper Palaeolithic dynamics. In M. Otte & F. Le Brun-Ricalens (Eds.), *Modes de Contacts et de Déplacements au Paléolithique Eurasiatique* (pp. 27–36). University of Liège Press.

[CR29] Belfer-Cohen, A., & Goring-Morris, A. N. (2017). The Upper Palaeolithic of Cisjordan. In Y. Enzel & O. Bar-Yosef (Eds.), *Quaternary of the Levant: Environments, climate change, and humans* (pp. 627–638). Cambridge University Press.

[CR30] Belfer-Cohen, A., & Goring-Morris, A. N. (2018). An anthropological review of the Upper Paleolithic in the Southern Levant. In A. Yasur-Landau, E. H. Cline, & Y. M. Rowan (Eds.), *The social archaeology of the Levant: From prehistory to the present* (pp. 29–46). Cambridge University Press.

[CR31] Belfer-Cohen, A., Davidzon, A., Goring-Morris, A. N., Lieberman, D., & Spears, M. (2004). Nahal Ein Gev I: A Late Upper Palaeolithic site by the Sea of Galilee, Israel. *Paléorient,**30*, 25–46.

[CR32] Bergman, C. A. (1988). Ksar Akil and the Upper Palaeolithic of the Levant. *Paléorient,**14*(2), 201–210.

[CR33] Bergman, C. A., & Stringer, C. B. (1989). Fifty years after: Egbert, an early Upper Palaeolithic juvenile from Ksar Akil, Lebanon. *Paléorient,**15*, 99–111.

[CR34] Bergman, C., Williams, J., Douka, K., & Schyle, D. (2017). The Palaeolithic sequence of Ksar ‘Akil, Lebanon. In Y. Enzel & O. Bar-Yosef (Eds.), *Quaternary of the Levant Environments, Climate Change, and Humans* (pp. 255–267). Cambridge University Press.

[CR35] Besançon, J., Copeland, L., & Hours, F. (1975). Tableaux de préhistoire libanaise. *Paléorient,**3*, 5–46.

[CR36] Boaretto, E., Hernandez, M., Goder-Goldberger, M., Aldeias, V., Regev, L., Caracuta, V., McPherron, S. P., Hublin, J. J., Weiner, S., & Barzilai, O. (2021). The absolute chronology of Boker Tachtit (Israel) and implications for the Middle to Upper Paleolithic transition in the Levant. *Proceedings of the National Academy of Sciences,**118*(25), e2014657118. 10.1073/pnas.2014657118 PMC8237571 34161257

[CR37] Bon, F. (2002). Les termes de l´ Aurignacien. *Espacio Tiempo y Forma Serie i, Prehistoria y Arqueología,**15*, 39–65.

[CR38] Bordes, J. G. (2006). News from the West: A reevaluation of the classical Aurignacian sequence of the Périgord. In O. Bar-Yosef & J. Zilhão (Eds.), *Towards a definition of the Aurignacian* (pp. 141–171). Instituto Portugues de Arqueologia.

[CR39] Bosch, M. D., Mannino, M. A., Prendergast, A. L., O’Connell, T. C., Demarchi, B., Taylor, S. M., Niven, L., Van Der Plicht, J., & Hublin, J. J. (2015). New chronology for Ksâr ‘Akil (Lebanon) supports Levantine route of modern human dispersal into Europe. *Proceedings of the National Academy of Sciences,**112*, 7683–7688. 10.1073/pnas.1501529112 PMC4485136 26034284

[CR40] Breuil, H. (1913). *Les subdivisions du paléolithique supérieur et leur signification*. Imprimerie Albert Kündig.

[CR41] Chiotti, L., Cretin, C., & Morala, A. (2015). The lithic industries from Blanchard and Castanet rock shelters (Dordogne, France): Data from the 2005–2012 excavations. *Palethnologie, Archéologie et Sciences Humaines, 7*, 76–97.

[CR42] Conard, N. J., & Bolus, M. (2008). Radiocarbon dating the late Middle Paleolithic and the Aurignacian of the Swabian Jura. *Journal of Human Evolution,**55*(5), 886–897. 18926559 10.1016/j.jhevol.2008.08.006

[CR43] Copeland, L. (1975). The Middle and Upper Paleolithic of Lebanon and Syria in the light of recent research. In F. Wendorf & A. E. Marks (Eds.), *Problems in prehistory: North Africa and the Levant* (pp. 317–350). Southern Methodist University Press.

[CR44] Copeland, L., & Hours, F. (1971). The later Upper Paleolithic material from Antelias Cave, Lebanon: Levels IV-I. *Berytus Archaeological Studies,**20*, 57–138.

[CR45] Davidzon, A., & Goring-Morris, A. N. (2003). Sealed in stone: The Upper Palaeolithic Early Ahmarian knapping method in the light of refitting studies at Nahal Nizzana XIII, Western Negev, Israel. *Journal of the Israel Prehistoric Society,**33*, 75–205.

[CR46] Davies, W. (2001). A very model of a modern human industry: New perspectives on the origins and spread of the Aurignacian in Europe. *Proceedings of the Prehistoric Society,**67*, 195–217.

[CR47] Dinnis, R., Bessudnov, A., Chiotti, L., Flas, D., & Michel, A. (2019). Thoughts on the structure of the European Aurignacian, with particular focus on Hohle Fels IV. *Proceedings of the Prehistoric Society,**85*, 29–60.

[CR48] Douka, K., Perlès, C., Valladas, H., Vanhaeren, M., & Hedges, R. E. M. (2011). Franchthi Cave revisited: The age of the Aurignacian in south-eastern Europe. *Antiquity,**85*(330), 1131–1150.

[CR49] Douka, K., Bergman, C. A., Hedges, R. E. M., Wesselingh, F. P., & Higham, T. F. G. (2013). Chronology of Ksâr ‘Akil (Lebanon) and implications for the colonization of Europe by Anatomically Modern Humans. *PLoS ONE,**8*, e72931. 24039825 10.1371/journal.pone.0072931 PMC3770606

[CR50] Ewing, J. F. (1947). Preliminary note on the excavations at the Palaeolithic site of Ksâr ‘Akil, Republic of Lebanon. *Antiquity,**21*(84), 186–196.

[CR51] Falcucci, A., Conard, N. J., & Peresani, M. (2020). Breaking through the Aquitaine frame: A re-evaluation on the significance of regional variants during the Aurignacian as seen from a key record in southern Europe. *Journal of Anthropological Sciences,**98*, 99–140. 33341757 10.4436/JASS.98021

[CR52] Ferring, C. R. (1976). Sde Divshon: An Upper Paleolithic site on the Divshon plain. In A. E. Marks (Ed.), *Prehistory and Paleoenvironments in the Central Negev, Israel* (Vol. II, pp. 99–206). Southern Methodist University Press.

[CR53] Ferring, C. R. (1988). Technological change in the Upper Paleolithic of the Negev. In H. Dibble & A. Montet-White (Eds.), *Upper Pleistocene prehistory of Western Eurasia* (pp. 333–348). University of Pennsylvania.

[CR54] Fox, J. R., & Coinman, N. R. (2004). Emergence of the Levantine Upper Paleolithic: Evidence from the Wadi al-Hasa. In P. J. Brantingham, S. L. Kuhn, & K. W. Kerry (Eds.), *The early Upper Paleolithic beyond Western Europe* (pp. 97–112). University of California Press.

[CR55] Garrod, D. A. E. (1934). The Stone Age of Palestine. *Antiquity,**8*(30), 133–150.

[CR56] Garrod, D. A. E. (1951). A transitional industry from the base of the Upper Palaeolithic in Palestine and Syria. *Journal of the Anthropological Institute of Great Britain and Ireland,**81*, 121–130.

[CR57] Garrod, D. A. E. (1953). The relations between south-west Asia and Europe in the later Palaeolithic age. *Journal of World History, 1*(1), 13–38.

[CR58] Garrod, D. A. E. (1955). The Mugharet el-Emireh in Lower Galilee: Type-station of the Emiran industry. *The Journal of the Royal Anthropological Institute of Great Britain and Ireland,**85*(1–2), 141–162.

[CR59] Garrod, D. A. E. (1957). Notes sur le Paléolithique Supérieur du Moyen Orient. *Bulletin De La Sociéte Préhistorique France,**55*, 239–445.

[CR60] Garrod, D. A. E., & Bate, D. M. A. (1937). The stone age of Mount Carmel. In *Excavations at the Wadi-Mughara* (Vol. 1). Clarendon Press.

[CR61] Ghazi, H. (2013). *Contribution à la connaissance de l’Aurignacien du Levant: analyse typo-technologique des industries lithiques de la séquence de Yabroud II (Syrie)*. University of Bordeaux 1.

[CR62] Gilead, I. (1981). The Upper Paleolithic tools assemblages from the Negev and Sinai. In J. Cauvin & P. Sanlaville (Eds.), *Prehistoire du Levant* (pp. 369–374). Centre National de la Recherche Scientifique.

[CR63] Gilead, I. (1991). The Upper Paleolithic period in the Levant. *Journal of World Prehistory,**5*, 105–154.

[CR64] Gilead, I. (1993). Upper Palaeolithic sites in the Ramat Matred area. *Palestine Exploration Quarterly,**125*(1), 19–42.

[CR65] Gilead, I., & Bar-Yosef, O. (1993). Early Upper Paleolithic sites in the Qadesh Barnea area, NE Sinai. *Journal of Field Archaeology,**20*(3), 265–280.

[CR66] Goder-Goldberger, M., & Malinsky-Buller, A. (2022). The Initial Upper Paleolithic and its place within the Middle-to-Upper Paleolithic transition of Southwest Asia: What hides behind the curtain of taxonomies? *Journal of Paleolithic Archaeology,**5*(1), 2.

[CR67] Goder-Goldberger, M., Crouvi, O., Caracuta, V., Horwitz, L. K., Neumann, F. H., Porat, N., Scott, L., Shavit, R., Jacoby-Glass, Y., Zilberman, T., & Boaretto, E. (2020). The Middle to Upper Paleolithic transition in the southern Levant: New insights from the late Middle Paleolithic site of Far’ah II, Israel. *Quaternary Science Reviews,**237*, 106304.

[CR68] Goder-Goldberger, M., Barzilai, O., & Boaretto, E. (2023). Innovative technological practices and their role in the emergence of Initial Upper Paleolithic technologies: A view from Boker Tachtit. *Journal of Paleolithic Archaeology,**6*(1), 11.

[CR69] González Echegaray, J. G. (1964). *Excavaciones en la terraza de “El Khiam”(Jordania): Estudio del yacimiento y los niveles paleolíticos*. Consejo Superior de Investigaciones Científicas.

[CR70] Goring-Morris, A. N. (1980). Upper Palaeolithic sites from Wadi Fazael, Lower Jordan Valley. *Paléorient,**6*, 173–191.

[CR71] Goring-Morris, A. N. (1987). *At the edge, terminal Pleistocene hunter-gatherers in the Negev and Sinai*. British Archaeological Reports.

[CR72] Goring-Morris, A. N. (1995). Upper Paleolithic occupation of the ‘Ein Qadis region on the Sinai/Negev border. *Atiqot,**27*, 1–14.

[CR73] Goring-Morris, A. N., & Belfer-Cohen, A. (2003). *More than meets the eye: Studies on Upper Paleolithic diversity in the Near East*. Oxbow Books.

[CR74] Goring-Morris, A. N., & Belfer-Cohen, A. (2017). The early and middle Epipaleolithic of Cisjordan. In Y. Enzel & O. Bar-Yosef (Eds.), *Quaternary of the Levant: Environments, climate change, and humans* (pp. 639–649). Cambridge University Press.

[CR75] Goring-Morris, A. N., & Belfer-Cohen, A. (2018). The Ahmarian in the context of the earlier Upper Palaeolithic in the Near East. In Y. Nishiaki & T. Akazawa (Eds.), *The Middle and Upper Paleolithic archeology of the Levant and beyond* (pp. 87–104). Springer.

[CR76] Goring-Morris, A. N., & Belfer-Cohen, A. (2020). Noisy beginnings: The Initial Upper Palaeolithic in southwest Asia. *Quaternary International,**551*, 40–46.

[CR77] Goring-Morris, A. N., & Davidzon, A. (2006). Straight to the point: Upper Paleolithic Ahmarian lithic technology in the Levant. *L’anthropologie,**44*(1), 93–111.

[CR78] Hershkovitz, I., Marder, O., Ayalon, A., Bar-Matthews, M., Yas’ur, G., Boaretto, E., Caracuta, V., Alex, B., Frumkin, A., Goder-Goldberger, M., Gunz, P., Holloway, R., Latimer, B., Lavi, R., Matthews, A., Slon, V., Bar-Yosef Mayer, D., Berna, F., Bar-Oz, G., et al. (2015). Levantine cranium from Manot Cave (Israel) foreshadows the first European modern humans. *Nature,**520*, 216e219. 25629628 10.1038/nature14134

[CR79] Hershkovitz, I., Weber, G. W., Quam, R., Duval, M., Grün, R., Kinsley, L., Ayalon, A., Bar-Matthews, M., Valladas, H., Mercier, N., & Arsuaga, J. L. (2018). The earliest modern humans outside Africa. *Science,**359*(6374), 456–459. 29371468 10.1126/science.aap8369

[CR80] Higham, T., Jacobi, R., Basell, L., Ramsey, C. B., Chiotti, L., & Nespoulet, R. (2011). Precision dating of the Palaeolithic: A new radiocarbon chronology for the Abri Pataud (France), a key Aurignacian sequence. *Journal of Human Evolution,**61*(5), 549–563. 21868058 10.1016/j.jhevol.2011.06.005

[CR81] Hours, F. (1974). Remarques sur l’utilisation de listes-types pour l’etude de Paleolithique Superieur et de l’Epipaleolithique du Levant. *Paléorient,**2*, 3–18.

[CR82] Hovers, E., Rak, Y., Lavi, R., & Kimbel, W. H. (1995). Hominid remains from Amud Cave in the context of the Levantine Middle Paleolithic. *Paléorient,**21*(2), 47–61.

[CR83] Jacobs, Z., Li, B., Jankowski, N., & Soressi, M. (2015). Testing of a single grain OSL chronology across the Middle to Upper Palaeolithic transition at Les Cottés (France). *Journal of Archaeological Science,**54*, 110–122.

[CR84] Jelinek, A. J. (1982). The Tabun Cave and Paleolithic man in the Levant. *Science,**216*(4553), 1369–1375. 17798344 10.1126/science.216.4553.1369

[CR85] Jones, M., Marks, A. E., & Kaufman, D. (1983). Boker: The artifacts. In A. E. Marks (Ed.), *Prehistory and Paleoenvironments in the Central Negev, Israel* (Vol. III, pp. 283–329). Southern Methodist University Press.

[CR86] Kadowaki, S. (2013). Issues of chronological and geographical distributions of Middle and Upper Palaeolithic cultural variability in the Levant and implications for the learning behavior of Neanderthals and Homo sapiens. In T. Akazawa, Y. Nishiaki, & K. Aoki (Eds.), *Dynamics of learning in Neanderthals and modern humans, volume 1: Cultural perspectives* (pp. 59–91). Springer Japan.

[CR87] Kadowaki, S., Omori, T., & Nishiaki, Y. (2015). Variability in Early Ahmarian lithic technology and its implications for the model of a Levantine origin of the Protoaurignacian. *Journal of Human Evolution,**82*, 67–87. 25924809 10.1016/j.jhevol.2015.02.017

[CR88] Kadowaki, S., Tamura, T., Sano, K., Kurozumi, T., Maher, L. A., Wakano, J. Y., Omori, T., Kida, R., Hirose, M., Massadeh, S., & Henry, D. O. (2019). Lithic technology, chronology, and marine shells from Wadi Aghar, southern Jordan, and Initial Upper Paleolithic behaviors in the southern inland Levant. *Journal of Human Evolution,**135*, 102646. 31450172 10.1016/j.jhevol.2019.102646

[CR89] Kuhn, S. L. (2004). From Initial Upper Paleolithic to Ahmarian at Üçağizli Cave, Turkey. *Anthropologie,**42*(3), 249–262.

[CR90] Kuhn, S. L. (2019). Initial Upper Paleolithic: A (near) global problem and a global opportunity. *Archaeological Research in Asia,**17*, 2–8.

[CR91] Kuhn, S. L., Stiner, M. C., Güleç, E., Özer, I., Yılmaz, H., Baykara, I., Açıkkol, A., Goldberg, P., Molina, K. M., Ünay, E., & Suata-Alpaslan, F. (2009). The Early Upper Paleolithic occupations at Üçağızlı Cave (Hatay, Turkey). *Journal of Human Evolution, 56*(2), 87–113. 19111331 10.1016/j.jhevol.2008.07.014

[CR92] Lengyel, G. (2007). *Lithic economy of the upper palaeolithic and epipalaeolithic of Raqefet Cave*. Oxford.

[CR93] Lengyel, G., Nadel, D., Tsatskin, A., Bar-Oz, G., Bar-Yosef Mayer, D. E., Be’eri, R., & Hershkovitz, I. (2005). Back to Raqefet Cave, Mount Carmel, Israel. *Journal of the Israel Prehistoric Society,**35*, 245–270.

[CR94] Lengyel, G., Boaretto, L., Fabre, L., & Ronen, A. (2006). New AMS 14 C dates from the Early Upper Paleolithic sequence of Raqefet Cave, Mount Carmel, Israel. *Radiocarbon,**48*, 253–258.

[CR95] Liolios, D. (1999). *Variabilité et caracteristiques du travail desmatières osseuses au debut de l'Aurignacien: Approche technologique et economique*. Université Paris X-Nanterre.

[CR96] Liolios, D. (2006). Reflections on the role of bone tools in the definition of the Early Aurignacian. In O. Bar-Yosef & J. Zilhão (Eds.), *Towards a definition of the Aurignacian* (pp. 37–51). Instituto Portugues de Arqueologia.

[CR97] Marder, O., Hershkovitz, I., & Barzilai, O. (2017). The Early Upper Paleolithic at Manot Cave, western Galilee: Chrono-cultural, subsistence and paleo-environmental reconstruction. In Y. Enzel & O. Bar-Yosef (Eds.), *Quaternary of the Levant Environments, Climate Change, and Humans* (pp. 277–284). Cambridge University Press.

[CR98] Marder, O., Barzilai, O., Abulafia, T., Hershkovitz, I., & Goder-Goldberger, M. (2018). Chrono-cultural considerations of Middle Paleolithic occurrences at Manot Cave (western Galilee), Israel. In Y. Nishiaki & T. Akazawa (Eds.), *The middle and upper Paleolithic Archeology of the Levant and Beyond* (pp. 49–63). Springer.

[CR99] Marder, O., Shemer, M., Abulafia, T., Bar-Yosef Mayer, D., Berna, F., Caux, S., Edeltin, L., Goder-Goldberger, M., Hershkovitz, I., Lavi, R., Shavit, R., Tejero, J.-M., Yeshurun, R., & Barzilai, O. (2021). Preliminary observations on the Levantine Aurignacian sequence of Manot Cave: Cultural affiliations and regional perspectives. *Journal of Human Evolution,**160*, 102705. 31882170 10.1016/j.jhevol.2019.102705

[CR100] Marks, A. E. (1976a). *Prehistory and Paleoenvironments in the Central Negev, Israel* (Vol. I). Southern Methodist University Press.

[CR101] Marks, A. E. (1976b). Ein Aqev: A late Levantine Upper Paleolithic site in the Nahal Aqev. In A. E. Marks (Ed.), *Prehistory and Paleoenvironments in the Central Negev, Israel* (Vol. I, pp. 227–291). Southern Methodist University Press.

[CR102] Marks, A. E. (1977a). *Prehistory and Paleoenvironments in the Central Negev, Israel* (Vol. II). Southern Methodist University Press.

[CR103] Marks, A. E. (1977b). The Upper Paleolithic sites of Boker Tachtit and Boker: A preliminary report. In A. E. Marks (Ed.), *Prehistory and Paleoenvironments in the Central Negev, Israel* (Vol. II, pp. 61–79). Southern Methodist University Press.

[CR104] Marks, A. E. (1981). The Upper Paleolithic of the Levant. In J. Cauvin & P. Sanlaville (Eds.), *Prehistoire du Levant* (pp. 369–374). Centre National de la Recherche Scientifique.

[CR105] Marks, A. E. (1983). *Prehistory and Paleoenvironments in the Central Negev, Israel* (Vol. III). Southern Methodist University Press.

[CR106] Marks, A. E. (2003). Reflections on Levantine Upper Palaeolithic studies: Past and present. In A. N. Goring-Morris & A. Belfer-Cohen (Eds.), *More than meets the eye: Studies on Upper Palaeolithic diversity in the Near East* (pp. 249–264). Oxbow.

[CR107] Marks, A. E., & Ferring, C. R. (1976). Upper Paleolithic sites near Ein Avdat. In A. E. Marks (Ed.), *Prehistory and Paleoenvironments in the Central Negev, Israel* (Vol. I, pp. 141–198). Southern Methodist University Press.

[CR108] Marks, A. E., & Ferring, C. R. (1988). The early Upper Paleolithic of the Levant. In J. F. Hoffecker (Ed.), *The early Upper Paleolithic: Evidence from Europe and the Near East* (pp. 43–72). Oxford.

[CR109] Marom, N., Lokshin-Gnezdilov, D., Shafir, R., Barzilai, O., & Shemer, M. (2022). Faunal remains from the Upper Paleolithic site of Nahal Rahaf 2 in the southern Judean Desert. *Peer Community Journal,**2*, e61.

[CR110] Meignen, L. (2012). Levantine perspectives on the Middle to Upper Paleolithic “transition.” *Archaeology, Ethnology and Anthropology of Eurasia,**40*(3), 12–21.

[CR111] Mellars, P. (2004). Neanderthals and the modern human colonization of Europe. *Nature,**432*(7016), 461–465. 15565144 10.1038/nature03103

[CR112] Mellars, P. (2006). Archeology and the dispersal of modern humans in Europe: Deconstructing the “Aurignacian.” *Evolutionary Anthropology: Issues, News, and Reviews,**15*(5), 167–182.

[CR113] Michel, A. (2010). *L'Aurignacien récent (post-ancien) dans le Sud-Ouest de la France: variabilité des productions lithiques, révision taphonomique et techno-économique des sites de Caminade-Est, abri Pataud, Roc-de-Combe, Le Flageolet I, La Ferrassie et Combemenue* (p. 1). Université Bordeaux.

[CR114] Michel, A. (2012). The production of flakes and laminar flakes during the middle Aurignacian: The case of layer 8 of the Pataud rock shelter (France). In A. Pastoors & M. Peresani (Eds.), *Flakes not blades—Discussing the role of flake making at the onset of the Upper Palaeolithic* (pp. 119–131). Wissenschaftliche Schriften des Neanderthal Museums.

[CR115] Monigal, K. (2002). *The Levantine Leptolithic: Blade production from the Lower Paleolithic to the dawn of the Upper Paleolithic*. Southern Methodist University.

[CR116] Nadel, D. (2003). The Ohalo II flint assemblage and the beginning of the Epipalaeolithic in the Jordan Valley. In A. N. Goring-Morris & A. Belfer-Cohen (Eds.), *More than meets the eye: Studies on Upper Palaeolithic diversity in the Near East* (pp. 216–229). Oxbow.

[CR117] Nadel, D., Carmi, I., & Segal, D. (1995). Radiocarbon dating of Ohalo II: Archaeological and methodological implications. *Journal of Archaeological Science,**22*(6), 811–822.

[CR118] Neuville, R. (1934). Le Préhistorique De Palestine. *Revue Biblique,**43*, 237–259.

[CR119] Neuville, R. (1951). *Le Paléolithique et le Mésolithique du Desert de Judée*. Archives de l’Institut de Paléontologie Humaine.

[CR120] Newcomer, M., & Watson, J. (1984). Bone artifacts from Ksar ‘Aqil (Lebanon). *Paléorient,**10*(1), 143–147.

[CR121] Nigst, P. R., Haesaerts, P., Damblon, F., Frank-Fellner, C., Mallol, C., Viola, B., Götzinger, M., Niven, L., Trnka, G., & Hublin, J. J. (2014). Early modern human settlement of Europe north of the Alps occurred 43,500 years ago in a cold steppe-type environment. *Proceedings of the National Academy of Sciences,**111*(40), 14394–14399. 10.1073/pnas.1412201111 PMC4209988 25246543

[CR122] Parow-Souchon, H. (2020). *The Wadi Sabra (Jordan), a contextual approach to the Palaeolithic landscape*. Verlag Marie Leidorf.

[CR123] Pelegrin, J. (1990). Prehistoric lithic technology: Some aspects of research. *Archeological Review from Cambridge,**9*, 116–125.

[CR124] Pelegrin, J. (1991). Les savoir-faire: Une très longue histoire. Terrain. *Anthropologie & Sciences Humaines,**16*, 106–113.

[CR125] Pelegrin, J. (2000). Les techniques de débitage laminaire au Tardiglaciaire: Critères de diagnose et quelques réflexions. L’Europe Centrale et Septentrionale au Tardiglaciaire. *Confrontation Des Modèles Régionaux,**7*, 73–86.

[CR126] Perrot, J. (1955). Le Paléolithique supérieur d’El Quseir et de Masaraq an Na’aj (Palestine) Inventaire de la collection René Neuville I et II. *Bulletin De La Société Préhistorique De France,**52*, 493–506.

[CR127] Phillips, J. L. (1988). The Upper Paleolithic of the Wadi Feiran, Southern Sinai. *Paléorient,**14*(2), 183–200.

[CR128] Rabinovich, R. (2003). The Levantine Upper Palaeolithic faunal record. In A. N. Goring-Morris & A. Belfer-Cohen (Eds.), *More than meets the eye: Studies on Upper Palaeolithic diversity in the Near East* (pp. 33–48). Oxbow.

[CR129] Rebollo, N. R., Weiner, S., Brock, F., Meignen, L., Goldberg, P., Belfer-Cohen, A., Bar-Yosef, O., & Boaretto, E. (2011). New radiocarbon dating of the transition from the Middle to the Upper Paleolithic in Kebara Cave, Israel. *Journal of Archaeological Science,**38*(9), 2424–2433.

[CR130] Ronen, A. (1984). *Sefunim prehistoric sites, Mount Carmel*. Israel.

[CR131] Rose, J. I., & Marks, A. E. (2014). “Out of Arabia” and the Middle-Upper Palaeolithic transition in the southern Levant. *Quartär,**61*, 49–85.

[CR132] Roussel, M., & Soressi, M. (2013). Une nouvelle séquence du Paléolithique supérieur ancien aux marges sud-ouest du Bassin parisien: Les Cottés dans la Vienne. In P. Bodu, L. Chehmana, L. Klaric, S. L. Mevel, & N. Teyssandier (Eds.), *Le Paléolithique supérieur ancien de l’Europe du nord-ouest. Réfléxions et synthèses à partir d’un projet collectif de recherche sur le centre et le sud du Bassin parisien* (pp. 283–298). Société Préhistorique Française.

[CR133] Schyle, D. (2015). The Levantine Aurignacian site of Sabra 4 – Palm view 1. In D. Schyle & J. Richter (Eds.), *Pleistocene archaeology of the Petra area in Jordan* (pp. 173–231). Gedruckt auf alterungsbeständigem.

[CR134] Schyle, D., & Richter, J. (2015). *Pleistocene Archaeology of the Petra Area in Jordan*. Gedruckt auf alterungsbeständigem.

[CR135] Schyle, D., & Uerpmann, H. P. (1988). Palaeolithic sites in the Petra area. In A. Garrard & H. G. Gebel (Eds.), *The prehistory of Jordan. The state of research in 1986* (pp. 39–65).

[CR136] Shemer, M., Boaretto, E., Greenbaum, N., Bar-Yosef Mayer, D., Tejero, J. M., Langgut, D., Gnezdilov, D. L., Barzilai, O., Marder, O., & Marom, N. (2023). Early Upper Paleolithic cultural variability in the Southern Levant: New evidence from Nahal Rahaf 2 Rockshelter, Judean Desert, Israel. *Journal of Human Evolution,**178*, 103342. 36934495 10.1016/j.jhevol.2023.103342

[CR137] Shemer, M., Barzilai, O., Boaretto, E., Hershkovitz, I., Lavi, R., Edeltin, L., & Marder, O. (2024). Intra-site variability – Analysis, characterization, and cultural affiliation of the Upper Paleolithic sequence of Manot Cave (western Galilee, Israel). *Archeological Research in Asia,**37*, 100501.

[CR138] Shimelmitz, R., Friesem, D. E., Clark, J. L., Groman-Yaroslavski, I., Weissbrod, L., Porat, N., & Kandel, A. W. (2018). The Upper Paleolithic and Epipaleolithic of Sefunim Cave, Israel. *Quaternary International,**464*, 106–125.

[CR139] Solecki, R. S. (1975). Shanidar IV, a Neanderthal flower burial in northern Iraq. *Science,**190*(4217), 880–881.

[CR140] Stringer, C. B., Grün, R., Schwarcz, H. P., & Goldberg, P. (1989). ESR dates for the hominid burial site of Es Skhul in Israel. *Nature,**338*(6218), 756–758. 2541339 10.1038/338756a0

[CR141] Tejero, J.-M., Yeshurun, R., Barzilai, O., Goder-Goldberger, M., Hershkovitz, I., Lavi, R., Schneller-Pels, N., & Marder, O. (2016). The osseous industry from Manot Cave (Western Galilee, Israel): Technological and conceptual behaviours of bone and antler exploitation in the Levantine Aurignacian. *Quaternary International,**403*, 90e106.

[CR142] Tejero, J.-M., Belfer-Cohen, A., Bar-Yosef, O., Gutkin, V., & Rabinovich, R. (2018). Symbolic emblems of the Levantine Aurignacians as a regional entity identifier (Hayonim Cave, Lower Galilee, Israel). *Proceedings of the National Academy of Sciences,**115*(20), 5145–5150. 10.1073/pnas.1717145115 PMC5960287 29712867

[CR143] Tejero, J.-M., Rabinovich, R., Yeshurun, R., Abulafia, T., Bar-Yosef, O., Barzilai, O., Belfer-Cohen, A., Goder-Golberger, M., Hershkovitz, I., Lavi, R., Shemer, M., & Marder, O. (2021). Personal ornaments from Hayonim and Manot Caves (Israel) hint to symbolic ties between the Levantine and the European Aurignacian. *Journal of Human Evolution,**160*, 102870. 32921424 10.1016/j.jhevol.2020.102870

[CR144] Teyssandier, N., & Zilhão, J. (2018). On the entity and antiquity of the Aurignacian at Willendorf (Austria): Implications for modern human emergence in Europe. *Journal of Paleolithic Archaeology,**1*, 107–138.

[CR145] Teyssandier, N., Bon, F., & Bordes, J. G. (2010). Within projectile range: Some thoughts on the appearance of the Aurignacian in Europe. *Journal of Anthropological Research,**66*(2), 209–229.

[CR146] Tostevin, G. B. (2012). *Seeing lithics: A middle-range theory for testing for cultural transmission in the Pleistocene*. Oxbow Books.

[CR147] Turville-Petre, F. (1927). Prehistoric Galilee. *Antiquity,**1*(3), 299–310.

[CR148] Turville-Petre, F. (1932). Excavations in the Mugharet el-Kebarah. *The Journal of the Royal Anthropological Institute of Great Britain and Ireland,**62*, 271–276.

[CR149] Valladas, H., Joron, J. L., Valladas, G., Arensburg, B., Bar-Yosef, O., Belfer-Cohen, A., Goldberg, P., Laville, H., Meignen, L., Rak, Y., & Tchernov, E. (1987). Thermoluminescence dates for the Neanderthal burial site at Kebara in Israel. *Nature,**330*(6144), 159–160.

[CR150] Vandermeersch, B. (1981). *Les hommes fossiles de Qafzeh*. Israël.

[CR151] Vandermeersch, B. (2007). Qafzeh, histoire des découvertes. *Bulletin Du Centre De Recherche Français à Jérusalem,**18*, 8–19.

[CR152] Vandermeersch, B., & Bar-Yosef, O. (2019). The Paleolithic burials at Qafzeh cave, Israel. *PALEO, Revue D’archéologie Préhistorique,**30–1*, 256–275.

[CR153] Williams, J. K. (2003). *Examining the boundaries of the Levantine Aurignacian*. Ph.D. Dissertation. Southern Methodist University.

[CR154] Williams, J. K. (2006). The Levantine Aurignacian: A closer look. In O. Bar-Yosef & J. Zilhão (Eds.), *Towards a definition of the Aurignacian* (pp. 317–352). Instituto Portugues de Arqueologia.

[CR155] Williams, J. K., & Bergman, C. (2010). Upper Paleolithic Levels XIII-VI (A and B) from the 1937–1938 and 1947–1948 Boston College Excavations and the ‘Levantine Aurignacian’ at Ksâr ‘Akil. *Lebanon. Paléorient,**36*(2), 117–161.

[CR156] Wurz, S., & Van Peer, P. (2012). Out of Africa, the Nile Valley and the Northern route. *The South African Archaeological Bulletin,**67*(196), 168–179.

[CR157] Yeshurun, R., Schneller-Pels, N., Barzilai, O., & Marder, O. (2021). Early Upper Paleolithic subsistence in the Levant: Zooarchaeology of the Ahmarian-Aurignacian sequence at Manot Cave, Israel. *Journal of Human Evolution,**160*, 102619. 31227173 10.1016/j.jhevol.2019.05.007

[CR158] Ziffer, D. (1981). Yabrud Shelter II. A re-consideration of its cultural composition and of its relevance to the Upper-Paleolithic cultural sequence in the Levant. *Quartär,**31*(32), 69–94.

[CR159] Zilhão, J., & d'Errico, F. (2006). The chronology of the Aurignacian and transitional technocomplexes. Where do we stand? In O. Bar-Yosef & J. Zilhão (Eds.), *Towards a definition of the Aurignacian* (pp. 313–349). Instituto Portugues de Arqueologia.

[CR160] Zumoffen, G. (1908). L’Age de la pierre en Phenicie. *Anthropos,**3*, 431–455.

